# Effects of Additive Incorporation on Fluoride Release of Orthodontic Glass Ionomer Cements and Adhesives: A Systematic Review

**DOI:** 10.3390/ma19163393

**Published:** 2026-08-10

**Authors:** Wojciech Dobrzyński, Sylwia Klimas, Zuzanna Majchrzak, Julia Kensy, Maja Gajewska, Anna Nikodem, Agata Małyszek, Maciej Dobrzyński, Jacek Matys, Marcin Mikulewicz

**Affiliations:** 1 Department of Dentofacial Orthopedics and Orthodontics, Division of Facial Abnormalities, Wroclaw Medical University, Krakowska 26, 50-425 Wroclaw, Poland; marcin.mikulewicz@umw.edu.pl; 2 Department of Pediatric Dentistry and Preclinical Dentistry, Wroclaw Medical University, Krakowska 26, 50-425 Wroclaw, Poland; sylwia.klimas@student.umw.edu.pl (S.K.); julia.kensy@student.umw.edu.pl (J.K.); maciej.dobrzynski@umw.edu.pl (M.D.); 3 Medical Center of Innovation, Wroclaw Medical University, Krakowska 26, 50-425 Wroclaw, Poland; zuzanna.h.nawrocka@gmail.com (Z.M.); m.gajewska97@gmail.com (M.G.); 4 Division of Biomedical Engineering and Experimental Mechanics, Wrocław University of Science and Technology, Smoluchowskiego 25, 50-370 Wroclaw, Poland; anna.nikodem@pwr.edu.pl; 5 Department of Biostructure and Animal Physiology, Wrocław University of Environmental and Life Sciences, Kożuchowska 1, 51-631 Wroclaw, Poland; agata.malyszek@upwr.edu.pl; 6 Oral Surgery Department, Wroclaw Medical University, Krakowska 26, 50-425 Wroclaw, Poland

**Keywords:** enamel demineralization, fluoride release, functional additives, glass ionomer cement, nanoparticles

## Abstract

This systematic review evaluated the effects of functional additives on fluoride release, fluoride recharge/re-release, and selected mechanical, physical, and biological properties of orthodontic glass ionomer cements (GICs) and glass ionomer-based adhesives. The review followed PRISMA guidelines. The protocol was retrospectively registered in the Open Science Framework. PubMed, Scopus, Web of Science, Embase, and WorldCat were searched for in vitro studies investigating modified orthodontic GICs and reporting fluoride-related or clinically relevant material properties. The final literature search was conducted on 30 April 2026. After screening 87 records, 23 studies were included in the qualitative synthesis. Owing to substantial methodological and outcome heterogeneity among the included studies, no meta-analysis was performed, and the findings were synthesized narratively. Risk of bias was assessed using the QUIN Tool; 20 studies were classified as having a medium risk of bias and three as having a low risk, while none were classified as having a high risk. The effects of additives on fluoride release varied according to additive type and concentration, material composition, storage conditions, pH, and evaluation period. Increases in fluoride release were reported in studies evaluating materials containing nCaF_2_, TMPnano, nanofluorapatite or nanofluorohydroxyapatite, hydroxyapatite, nanochitosan, SNP/ZnONP, and ZnSO_4_. Several additives maintained or improved mechanical performance, whereas higher concentrations of some agents adversely affected bond strength, microhardness, or material stability. Antibacterial and antibiofilm activity was most evident for CHX, DMAHDM, MDPB, propolis, silibinin, nanosilver, and zinc-containing systems. Limited evidence also suggested potential benefits regarding enamel protection and cytocompatibility. Functional additives may enhance the preventive potential of orthodontic GIC-based materials, particularly through improved fluoride release and antibacterial activity. However, further standardized long-term and clinical studies are required before clinical recommendations can be made.

## 1. Introduction

Orthodontic treatment enables patients with malocclusions to improve both smile esthetics and masticatory function, thereby contributing to oral health-related quality of life. However, treatment with fixed appliances is often long-lasting and may considerably impair oral hygiene. Brackets, archwires, ligatures, and orthodontic bands promote the retention of dental plaque and food debris, creating multiple areas that are difficult to clean [1]. Brackets may hinder effective cleaning of the cervical and labial enamel surfaces, while ligatures can limit interdental hygiene and flossing. In addition, gaps between orthodontic bands and tooth surfaces may favor food accumulation and biofilm formation. In patients with insufficient oral hygiene control during long-term treatment, gingival overgrowth around brackets may further impair plaque removal [2].

**Figure 1:**
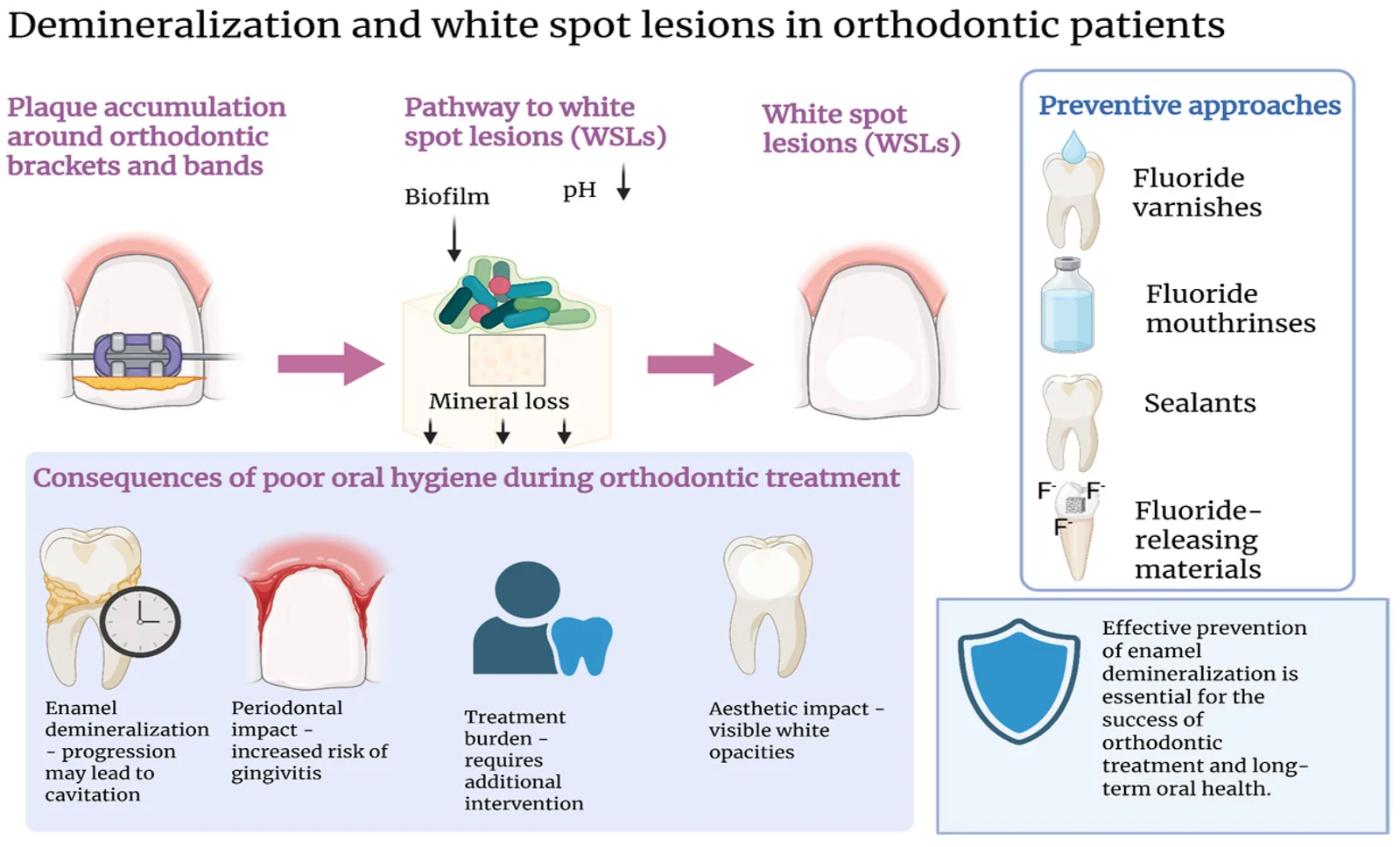
Demineralization and white spot lesions in orthodontic patients.

White spot lesions are among the most common complications associated with fixed orthodontic treatment. They usually develop around brackets and bonding materials as a result of prolonged plaque accumulation and acid production by cariogenic microorganisms [3]. A decrease in oral pH promotes the loss of calcium and phosphate ions from enamel, leading to demineralization [4]. At an early stage, this process may be arrested or partially reversed through remineralization. However, if poor oral hygiene and bacterial activity persist, enamel demineralization may progress to cavitation and dentin involvement, requiring invasive restorative treatment. Because multiple teeth are usually covered with fixed orthodontic appliances, these lesions may occur simultaneously in several areas and compromise the final esthetic outcome of orthodontic therapy. Therefore, preventive strategies aimed at maintaining enamel integrity are essential during fixed appliance treatment. Common approaches include fluoride mouthwashes, fluoride varnishes, protective sealants, and fluoride-releasing materials [5,6,7,8,9,10,11] (Figure 1).

Glass ionomer cements are widely used in orthodontics for cementing bands and, in selected cases, for bonding components of fixed appliances, including brackets. The main materials used for these purposes include conventional glass ionomer cements and resin-modified glass ionomer cements. These materials can chemically adhere to enamel, dentin, and metal, reducing the need for aggressive enamel conditioning compared with composite resin systems [12,13,14,15]. One of their most important advantages is their ability to release fluoride ions, especially during the initial period after application [11,15,16,17,18,19,20,21,22]. Fluoride release may contribute to anticaries and remineralizing effects by reducing enamel demineralization around orthodontic appliances. Some glass ionomer materials can also be recharged with fluoride from external sources, such as fluoride mouthwashes or varnishes, and subsequently re-release fluoride over time [10,19,21,23,24,25,26,27,28,29]. This mechanism may support the formation of fluorohydroxyapatite or fluoroapatite, which are more resistant to acidic conditions. Resin-modified glass ionomer cements generally offer improved mechanical strength, shorter setting time, and better moisture tolerance than conventional formulations, while maintaining fluoride-releasing capacity [15,21,22,30,31]. Nevertheless, glass ionomer-based materials still have limitations, including lower mechanical performance than resin composites and susceptibility to environmental factors such as pH changes, saliva, water sorption, and masticatory forces. Despite these limitations, their fluoride release, chemical adhesion, and preventive potential make them valuable materials in orthodontic treatment.

Orthodontic glass ionomer cements and glass ionomer-based adhesive systems can be modified by incorporating functional additives that may influence their chemical, mechanical, and biological properties [12,32,33,34,35,36,37,38,39,40,41,42,43,44,45]. These additives include fluoride-releasing and remineralizing compounds, natural substances, biopolymers, antibacterial agents, and nanoparticles. Fluoride-containing compounds, such as nano-calcium fluoride, sodium fluoride, nano-fluorapatite, and nano-fluorohydroxyapatite, may enhance fluoride release and recharge capacity, thereby increasing the cariostatic potential of orthodontic materials and improving protection against enamel demineralization [17,19,42,44,46,47,48,49,50,51]. Remineralizing additives, including amorphous calcium phosphate, bioactive glass, hydroxyapatite, seashell-derived nanoparticles, and sodium trimetaphosphate, may promote the release of beneficial ions and support enamel remineralization [7,35,36,37,39,41,45,50,51,52,53,54]. Natural substances and biopolymers, such as chitosan [32,44,45,55,56,57], phosphorylated chitosan, red propolis extract [58], and silibinin, have also shown promising antibacterial or antibiofilm effects [59]. Other bioactive agents, including graphene, silver nanoparticles, zinc oxide nanoparticles, and zinc-containing compounds, may reduce bacterial viability and biofilm formation [33,40,43,45,56,60,61,62,63,64,65,66]. However, the incorporation of additives may simultaneously affect several clinically relevant material properties, including fluoride release, fluoride recharge capacity, bond strength, microhardness, surface quality, cytotoxicity, and antibacterial efficacy [30,31,38,39,40,45,67,68,69,70,71]. Therefore, optimization of additive type and concentration is essential to obtain a balanced material that combines bioactivity with acceptable mechanical stability and biological safety.

The aim of this systematic review was to evaluate the influence of functional additives on fluoride release, fluoride recharge/re-release capacity, and selected mechanical, physical, and biological properties of orthodontic glass ionomer cements and orthodontic glass ionomer-based adhesive materials. Attention was given to fluoride-releasing and remineralizing agents, natural and biopolymeric additives, and antibacterial nanoparticles intended to enhance the preventive and bioactive performance of materials used in orthodontic therapy. Fluoride release from these materials is of clinical relevance, as it may contribute to enamel remineralization and caries prevention during fixed orthodontic treatment with brackets, where patients are at increased risk of demineralization. This review also considered the effects of these modifications on clinically relevant parameters in orthodontics, including bond strength, hardness, cytocompatibility, antibacterial activity, and enamel-protective potential. Previous reviews have addressed fluoride-releasing restorative materials broadly [11], factors influencing fluoride release from specific material categories, such as metal-modified glass ionomer cements [72], or the clinical effectiveness of fluoride-containing orthodontic adhesives in preventing enamel decalcification [73]. However, these reviews did not comprehensively evaluate the experimental incorporation of chemically diverse functional additives into orthodontic glass ionomer cements and glass ionomer-based adhesives. In contrast, the present systematic review integrates fluoride release and recharge/re-release outcomes with mechanical and adhesive properties, physicochemical characteristics, antibacterial and antibiofilm activity, cytocompatibility, and enamel-protective effects. This combined assessment addresses an important knowledge gap because an additive that improves fluoride delivery or antimicrobial activity may simultaneously compromise bond strength, hardness, surface stability, setting behavior, or biological safety. To the best of the authors’ knowledge, no previous systematic review has evaluated these potential benefits and trade-offs together across multiple classes of functional additives specifically incorporated into orthodontic glass ionomer-based materials.

## 2. Materials and Methods

### 2.1. Focused Question

The systematic review followed the PICO framework as follows: in orthodontic glass ionomer cements and glass ionomer-based adhesives (population), does the incorporation of functional additives (intervention) improve fluoride release and/or fluoride recharge capacity (outcome) compared with unmodified materials or control formulations without additives (comparison)?

### 2.2. Protocol

**Figure 2:**
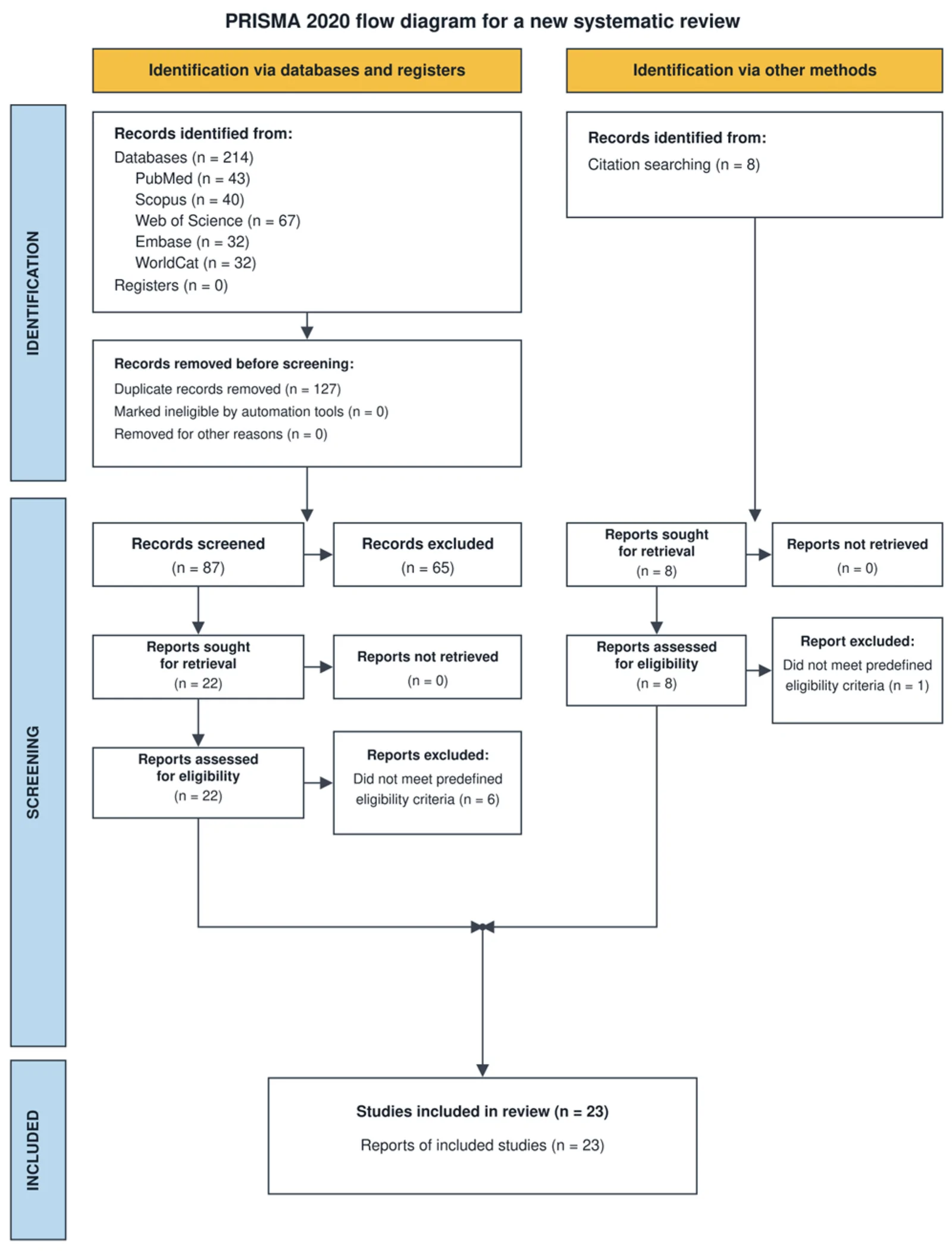
The PRISMA 2020 flow diagram [74,75].

The selection process for articles in the systematic review was meticulously outlined according to the PRISMA flow diagram (Figure 2) [74,75] (Supplementary Materials). The systematic review was retrospectively registered with the Open Science Framework under the following link: https://osf.io/hwc2u/overview, after completion of the literature search, study screening, and data extraction phases (assessed on 8 June 2026).

### 2.3. Eligibility Criteria

Studies were considered eligible for inclusion if they fulfilled the following criteria [72,76,77,78,79,80,81,82,83,84,85,86]:

- Studies evaluating orthodontic glass ionomer cements, resin-modified glass ionomer cements, or glass ionomer-based orthodontic adhesives with incorporated functional additives;
- Studies assessing fluoride release, fluoride recharge/re-release, or material properties relevant to orthodontic applications;
- In vitro studies;
- Studies including an unmodified material, commercially available material, or other appropriate control group;
- studies published in English.

The following exclusion criteria were applied [72,76,77,78,79,80,81,82,83,84,85,86]:

- Studies not evaluating the incorporation of additives into orthodontic glass ionomer cements or glass ionomer-based adhesives;
- Studies focusing exclusively on non-orthodontic restorative materials;
- Studies not reporting fluoride-related outcomes or clinically relevant mechanical, physical, biological, antibacterial, cytotoxicity, remineralization, or enamel-protective outcomes;
- Studies without a control group;
- Non-English papers;
- reviews, systematic reviews, meta-analyses, conference abstracts, editorials, letters, and opinion articles;
- Studies with no full text available;
- Duplicate publications.
- No restrictions were applied regarding the year of publication.

The eligibility criteria were defined a priori according to the focused PICO question and the predefined review protocol. The methodological framework and reporting approach are consistent with that reported in previous systematic reviews; however, these publications did not determine the present eligibility criteria [72,76,77,78,79,80,81,82,83,84,85,86].

### 2.4. Information Sources, Search Strategy, and Study Selection

An extensive literature search was performed in PubMed, Scopus, Embase, and Web of Science (WoS), and was supplemented by a gray literature search in WorldCat. The search was conducted up to April 2026 to identify studies that met the predefined eligibility criteria. The search strategy focused on orthodontic glass ionomer cements and glass ionomer-based adhesives modified with additives, with particular emphasis on fluoride release and fluoride recharge. Searches were limited to titles and abstracts, and controlled vocabulary or subject headings were used where applicable.

The search strategy used for each database was as follows:

PubMed: orthodontic* AND (“glass ionomer cement*” OR “glass ionomer adhesive*” OR “resin modified glass ionomer*” OR RMGIC) AND (modif* OR addit* OR incorporat* OR dop* OR nanoparticle*) AND (“fluoride release” OR “fluoride recharge”).

Scopus: TITLE-ABS-KEY (orthodontic* AND (“glass ionomer cement*” OR “glass ionomer adhesive*” OR “resin modified glass ionomer*” OR RMGIC) AND (modif* OR addit* OR incorporat* OR dop* OR nanoparticle*) AND (“fluoride release” OR “fluoride recharge”)).

Embase: (orthodontic*:ti,ab AND (“glass ionomer cement*” OR “glass ionomer adhesive*” OR “resin modified glass ionomer*” OR RMGIC):ti,ab AND (modif* OR addit* OR incorporat* OR dop* OR nanoparticle*):ti,ab AND (“fluoride release” OR “fluoride recharge”):ti,ab).

Web of Science: TS = (orthodontic* AND (“glass ionomer cement*” OR “glass ionomer adhesive*” OR “resin modified glass ionomer*” OR RMGIC) AND (modif* OR addit* OR incorporat* OR dop* OR nanoparticle*) AND (“fluoride release” OR “fluoride recharge”)).

WorldCat: orthodontic* AND “glass ionomer cement*” AND (“fluoride release” OR “fluoride recharge”) AND (modif* OR addit* OR nanoparticle*).

Following the search, all identified records were imported into a reference management file, duplicates were removed, and the remaining records were screened by title and abstract. Potentially eligible articles were then assessed in full text according to the predefined inclusion and exclusion criteria. Any disagreements between reviewers were resolved through discussion until consensus was reached.

### 2.5. Data Collection Process and Data Items

The study selection and data extraction processes were conducted independently by five reviewers (W.D., J.K., S.K., M.G., and Z.M.). Each reviewer assessed every record during the title and abstract screening and full-text eligibility assessment; the records were not divided among reviewer pairs. All five reviewers also independently extracted data from each eligible study. The predefined data items included the first author, year of publication, study design, article title, type of orthodontic glass ionomer cement or adhesive, incorporated additive, control material, fluoride release or recharge outcomes, and other relevant mechanical, physical, or biological properties. After completion of the independent assessments, the reviewers compared their findings. Any discrepancies were addressed by re-examining the original article and the predefined eligibility or data extraction criteria, followed by discussion until unanimous consensus was reached. The final consensus dataset was entered into a predesigned electronic spreadsheet prepared in Microsoft Excel 365 (Version 2505, Build 16.0.18827.20102, 64-bit), ensuring consistent and systematic data recording.

### 2.6. Risk of Bias and Quality Assessment

The methodological quality and risk of bias of the included studies were independently assessed by two reviewers (W.D. and J.M.) using the QUIN Tool [87]. Because the assessment required access to the full texts, the reviewers were not blinded to the study authors, journals, or reported results. However, each reviewer completed the initial QUIN assessment independently and without access to the other reviewer’s ratings. Their assessments were compared only after both evaluations had been completed. Disagreements concerning individual QUIN items were resolved by re-examining the relevant study and discussing the assessments until consensus was reached.

Because W.D. was involved in the authorship of study [88], the methodological quality of this study was reassessed independently by three reviewers (J.M., J.K., and S.K.), none of whom were involved in its authorship. The reviewers applied the same QUIN criteria as those used for all other included studies and completed their initial assessments without access to one another’s ratings. Any disagreements were resolved by re-examining the study and discussing the assessments until consensus was reached. Study [88] was excluded from the two-reviewer Cohen’s kappa calculation.

The QUIN Tool comprises 12 criteria covering the principal methodological domains of in vitro studies. Each applicable item was scored as “adequately specified” (score = 2), “inadequately specified” (score = 1), or “not specified” (score = 0). For each study, the final QUIN score was calculated using the following formula:

Final score (%) = (total score obtained × 100)/(2 × number of applicable criteria).

Based on the final percentage score, studies were categorized as having a low risk of bias (>70%), medium risk of bias (50–70%), or high risk of bias (<50%).

Agreement between the initial, pre-consensus QUIN assessments conducted by W.D. and J.M., excluding study [88], was evaluated using Cohen’s kappa coefficient. The calculation was performed using MedCalc software (version 23.1.7; MedCalc Software Ltd., Ostend, Belgium). Cohen’s kappa was 0.84 (*p* < 0.001), indicating almost perfect agreement between the two reviewers. Most included studies were classified as having a medium risk of bias, whereas only three studies were categorized as having a low risk of bias.

## 3. Results

### 3.1. Study Selection

A total of 214 records were identified through database and gray literature searching: 43 from PubMed, 40 from Scopus, 67 from Web of Science, 32 from Embase, and 32 from WorldCat. After removal of 127 duplicate records, 87 records remained for title and abstract screening. During screening, studies were considered potentially eligible if they investigated orthodontic glass ionomer cements or glass ionomer-based adhesives modified by the incorporation of active substances, fillers, nanoparticles, or other functional additives. Studies were also required to report fluoride release, fluoride recharge/re-release, or material-related outcomes relevant to orthodontic applications.

Records that did not address additive-modified orthodontic glass ionomer-based materials or did not report fluoride-related or material-performance outcomes were excluded. Six full-text articles were excluded because they did not meet the predefined inclusion criteria. One study evaluated a composite cement paste rather than a glass ionomer-based orthodontic material [89]. Another study was excluded because no additive was incorporated into the examined cements [90]. One article was not focused on fluoride release [91], while another did not assess additive incorporation or fluoride-release measurements [92]. Two further studies were excluded because the tested materials did not include incorporated additives [93,94].

After title and abstract screening, 22 full-text articles were assessed for eligibility. After completion of the database-derived selection process, 16 studies fulfilled the predefined eligibility criteria. Subsequent hand-searching of the reference lists of the included articles yielded eight additional relevant studies. Consequently, 23 studies were included in the final qualitative synthesis of this systematic review [42,44,45,51,88,95,96,97,98,99,100,101,102,103,104,105,106,107,108,109,110,111,112].

### 3.2. General Characteristics of the Included Studies

The included studies were consistent with the scope of this systematic review, as they assessed the influence of additive incorporation on fluoride release, fluoride recharge/re-release, and other relevant properties of orthodontic glass ionomer cements and glass ionomer-based adhesives.

The included articles were published between 2003 and 2025. The evaluated materials comprised conventional glass ionomer cements, resin-modified glass ionomer cements, orthodontic glass ionomer adhesives, and experimental fluoride-releasing orthodontic cement systems [42,44,45,51,88,95,96,97,98,99,100,101,102,103,104,105,106,107,108,109,110,111,112]. Most studies compared experimentally modified materials with unmodified control formulations or commercially available orthodontic cements and adhesives.

The incorporated additives varied considerably in terms of origin, composition, and intended function. Several studies evaluated naturally derived or polymer-based additives, including graphene [95], red propolis extract [96], chitosan or phosphorylated chitosan [97,98], silibinin [103], and nanochitosan [44]. Other studies focused on inorganic, mineral, or bioactive additives, such as seashell-derived nanoparticles [42], nano-calcium fluoride [99,106], nano-fluorapatite or nano-fluorohydroxyapatite [88,102], silver–hydroxyapatite–silica hybrid nanoparticles [104], eggshell powder [51], hydroxyapatite derived from marine sources [110], CPP-ACP, bioactive glass, and MDPB [45]. Further modifications included chlorhexidine, nano-sized sodium trimetaphosphate, chlorhexidine-modified halloysite nanotubes, nanosilver, zinc oxide nanoparticles, zinc sulfate, and multifunctional monomer or nanoparticle systems such as DMAHDM, MPC, and NACP [105,106,107,108,109,111,112].

Fluoride release was one of the principal outcomes assessed across the included studies, although the duration and methodology of fluoride measurement differed substantially. Some studies evaluated fluoride release over short periods, ranging from 24 h to several days [44,45,96], whereas others assessed fluoride release over 1–4 weeks [42,51,95,98,109,111,112] or over longer observation periods lasting several weeks to months [88,99,100,101,102,110]. Several studies also investigated fluoride recharge or re-release capacity, particularly in materials modified with nano-calcium fluoride, nanofluorapatite, silver–hydroxyapatite–silica nanoparticles, silibinin, or zinc-containing additives [88,99,103,104,112]. These differences in observation period, storage medium, and analytical method should be considered when comparing fluoride-related outcomes across studies.

In addition to fluoride-related outcomes, the included studies frequently assessed mechanical, adhesive, and physicochemical properties. Shear bond strength was examined in studies evaluating red propolis-modified GIC, chitosan-modified RMGIC, nano-calcium fluoride-containing cement, nanosonosensitizer-modified RMGIC, nanofluorapatite-modified RMGIC, silibinin-modified RMGIC, and nanosilver-containing orthodontic cements [96,97,99,101,102,103,108]. Other commonly reported mechanical outcomes included compressive strength, flexural strength, diametral tensile strength, microhardness, surface hardness, wear resistance, and setting time [42,44,45,51,95,96,98,102,104,105,109,110,111,112]. Selected studies also examined additional material characteristics, including surface roughness, radiopacity, water sorption, solubility, color stability, surface morphology, degree of conversion, and structural changes assessed by microscopic or spectroscopic methods [42,95,98,102,104,110,111,112].

Antimicrobial and antibiofilm properties represented another important area of investigation. Most antimicrobial analyses focused on *Streptococcus mutans*, reflecting its relevance to enamel demineralization and white spot lesion development during orthodontic treatment [45,96,98,101,103,105,106,107,108,109,112]. Some studies also assessed other microorganisms, including *Candida albicans* and *Lactobacillus acidophilus* [45,96]. The antimicrobial strategies investigated included natural extracts, biopolymeric systems, antibacterial monomers, chlorhexidine-based systems, nanosilver, zinc-containing additives, MDPB, and ultrasound-activated nanosonosensitizers [45,96,98,101,103,105,106,107,108,109,112].

A smaller group of studies investigated biological safety or enamel-related outcomes. Cytotoxicity or cell viability was assessed in studies evaluating TMPnano/phosphorylated chitosan-modified RMGIC, nano-calcium fluoride-containing orthodontic cement, silibinin-modified RMGIC, and nCaF_2_/DMAHDM-modified RMGIC [98,99,103,106]. Enamel protection, demineralization inhibition, lesion depth, enamel hardness, or remineralization potential were assessed in studies incorporating nCaF_2_, DMAHDM, MPC, NACP, chlorhexidine, and TMP-based systems [99,106,107,109]. Overall, substantial methodological heterogeneity was observed among the included studies, particularly with respect to material type, additive concentration, experimental protocol, storage medium, follow-up duration, and outcome assessment method.

A general overview of the included studies is presented in Table 1, while detailed study characteristics are summarized in Table 2.

### 3.3. Main Study Outcomes

To summarize the main findings of the included studies, detailed study outcomes are presented in Table 2.

#### 3.3.1. Fluoride Release and Fluoride Recharge

Fluoride release was assessed in most of the included studies and represented the main fluoride-related outcome of this review. The reported effects were heterogeneous and depended on the type and concentration of the additive, base material composition, storage medium, pH conditions, analytical method, and observation period.

An increase in fluoride release was most often reported for materials modified with fluoride-, calcium-, phosphate-, apatite-, chitosan-, or selected metal nanoparticle-based additives. These included nCaF_2_-containing formulations [99,106], TMPnano with or without phosphorylated chitosan [98], nano-fluorapatite/nano-fluorohydroxyapatite or nanofluorapatite [88,102], Ag/HA/Si hybrid nanoparticles [104], CHX/TMP-containing formulations [109], marine-derived hydroxyapatite [110], nanochitosan [44], combined silver/zinc oxide nanoparticles [111], and ZnSO_4_ [112]. Increased fluoride release was also observed under specific experimental conditions after incorporation of graphene [95], chitosan [97], seashell-derived nanoparticles [42], and selected CPP-ACP-, BAG-, CH-, or MDPB-modified GICs, with BAG-GIC showing the highest early fluoride release in that comparative study [45]. Chicken eggshell powder improved selected mechanical properties, while its overall effect on fluoride release was limited or not clearly pronounced [51].

In contrast, some additives did not produce a sustained or clinically meaningful improvement in fluoride release [96,101,103]. Red propolis extract did not significantly affect fluoride release despite improving antimicrobial activity [96]. Ultrasound-activated nanosonosensitizers did not significantly change fluoride release compared with the control, although they enhanced antibiofilm activity after activation [101]. Silibinin-modified RMGIC did not substantially alter the overall fluoride release pattern, despite differences observed at selected time points [103]. Studies evaluating fluoride-releasing bracket systems or commercially available orthodontic GICs without experimental additive incorporation were considered mainly as comparative background evidence for fluoride-release kinetics [100].

Several studies showed an initial burst of fluoride release followed by a gradual decline or stabilization phase, particularly in conventional or resin-modified glass ionomer-based materials [88,100,102,109,112]. Fluoride recharge or re-release was assessed less frequently than fluoride release, with relevant evidence reported particularly for nCaF_2_-containing orthodontic cement [99], nanofluorapatite-modified RMGIC [88], Ag/HA/Si-containing materials [104], silibinin-modified RMGIC [103], and ZnSO_4_-modified GICs [112]. Overall, additive incorporation may enhance fluoride delivery, but this effect was material-specific, concentration-dependent, and not consistent across all modified orthodontic glass ionomer cements and adhesives.

#### 3.3.2. Mechanical and Physical Properties

Mechanical, adhesive, and physical properties were assessed in several included studies, mainly by shear bond strength, compressive strength, flexural strength, diametral tensile strength, microhardness, surface roughness, setting time, solubility, radiopacity, porosity, wear resistance, and color stability [42,44,45,51,95,96,97,98,99,101,102,103,104,105,106,107,108,109,110,111,112]. The effect of additive incorporation was not uniform.

Improvement or preservation of selected mechanical properties was reported for several additives. Graphene increased microhardness at lower concentrations and reduced surface roughness, although it decreased radiopacity [95]. Chitosan preserved the adhesive bond strength of RMGIC [97]. TMPnano combined with 0.25% phosphorylated chitosan showed the most favorable mechanical profile among the tested TMPnano/Chi-Ph formulations [98]. Seashell-derived nanoparticles improved compressive strength and microhardness [42]. Chicken eggshell powder improved compressive strength and microhardness, while maintaining ion-release behavior [51]. Nanochitosan increased compressive strength, flexural strength, and wear resistance [44]. Ag/HA/Si hybrid nanoparticles did not significantly affect compressive strength despite increasing fluoride release at higher concentrations [104]. Red propolis extract modified the antimicrobial profile of orthodontic GICs, while the 25% concentration had the least negative influence on mechanical properties and did not significantly alter fluoride release [96].

For orthodontic bonding, the effects of additive incorporation on shear bond strength were concentration-dependent and varied among studies. No universal numerical threshold was prespecified or applied to determine clinical acceptability. Because the experimental protocols differed in tooth substrate and preparation, bracket-base characteristics, storage and aging conditions, crosshead speed, loading configuration, and test geometry, absolute SBS values were interpreted primarily in relation to the corresponding control group within each study. nCaF_2_-containing experimental cements showed bond strength comparable to commercial RMGIC controls under the conditions used [99]. The formulation containing 20% nCaF_2_ and 3% DMAHDM showed bond strength comparable to the unmodified GC Ortho LC control, whereas 30% nCaF_2_ and 4% DMAHDM reduced SBS [106]. MPC/DMAHDM/NACP-modified RMGIC showed bond strength comparable to unmodified RMGIC but lower than Transbond XT [107]. Nanosilver-containing cements showed SBS values of approximately 6–8 MPa under the specific experimental conditions used, but bond strength decreased as nanosilver concentration increased and the 15% nanosilver formulation showed significantly lower SBS than the control [108]. Nano-fluorapatite and nano-fluorohydroxyapatite increased fluoride release but reduced shear and micro-tensile bond strength relative to unmodified Fuji Ortho LC [102]. Silibinin [103] and ultrasound-activated nanosonosensitizers [101] also produced concentration-dependent reductions in SBS. These findings should be interpreted as comparative in vitro mechanical outcomes rather than evidence of clinical suitability.

For orthodontic bonding, acceptable shear bond strength was generally maintained only within specific additive ranges. nCaF_2_-containing experimental cements maintained bond strength comparable to commercial RMGIC controls [99], whereas RMGICs containing nCaF_2_ and DMAHDM preserved enamel bond strength only at optimized concentrations; higher concentrations, such as 30% nCaF_2_ or 4% DMAHDM, reduced bonding performance [106]. MPC/DMAHDM/NACP-modified RMGIC showed bond strength comparable to unmodified RMGIC, but lower than Transbond XT [107]. Nanosilver-containing orthodontic cements remained within clinically acceptable ranges, although shear bond strength decreased as nanosilver concentration increased [108]. Similarly, nano-fluorapatite and nano-fluorohydroxyapatite increased fluoride release but reduced shear and micro-tensile bond strength compared with unmodified Fuji Ortho LC [102]. Silibinin [103] and ultrasound-activated nanosonosensitizers [101] also caused concentration-dependent reductions in shear bond strength, although selected formulations remained clinically acceptable.

CHX/TMP-modified RMGICs showed reduced compressive and tensile strength at higher additive concentrations [109]. Marine-derived hydroxyapatite increased surface roughness in both conventional and resin-modified GICs [110]. Combined SNP/ZnONP incorporation increased fluoride release but was associated with lower microhardness and greater porosity [111]. ZnSO_4_ did not significantly affect flexural strength, although solubility increased with additive concentration while remaining within acceptable limits [112]. Overall, the available evidence indicates that additive incorporation may improve or preserve selected material properties, but mechanical outcomes are strongly concentration-dependent, and excessive additive loading may compromise bond strength, surface quality, or matrix integrity.

#### 3.3.3. Antibacterial and Antibiofilm Activity

Antibacterial or antibiofilm activity was assessed in a subset of the included studies, most commonly against *Streptococcus mutans*, with selected studies also including *Candida albicans* or *Lactobacillus acidophilus* [45,96,98,101,103,105,106,108,109,112]. Red propolis extract reduced the viability of *S. mutans* and *C. albicans*, particularly at higher concentrations, without significantly altering fluoride release [96]. Silibinin-modified RMGIC reduced *S. mutans* counts and biofilm formation in a concentration-dependent manner, with the strongest effect observed at 5%, although chlorhexidine remained more effective [103]. TMPnano combined with phosphorylated chitosan reduced *S. mutans* viability and biofilm metabolism while maintaining acceptable cytocompatibility [98].

More targeted antimicrobial systems showed stronger but often formulation-dependent effects [45,105,106,109]. Chlorhexidine-modified halloysite nanotubes inhibited *S. mutans*, particularly at 10%; however, the durability of this antibacterial effect was not clearly established in long-term testing [105]. CHX/TMP-modified RMGICs reduced biofilm metabolism and showed antibacterial activity, but higher additive concentrations negatively affected mechanical properties [109]. DMAHDM-containing RMGIC reduced biofilm viability, metabolic activity, polysaccharide production, lactic acid production, and CFU counts, with the nCaF_2_/DMAHDM formulation showing a pronounced antibiofilm effect among the tested groups [106]. MDPB-modified GIC showed the lowest bacterial adhesion against *S. mutans* and *L. acidophilus* among CPP-ACP-, BAG-, chitosan-, and MDPB-modified materials [45].

Nanoparticle- and ion-based antimicrobial strategies showed variable durability. Ultrasound-activated nanosonosensitizers significantly reduced *S. mutans* biofilm after activation, with 5% nano-emodin showing a favorable balance between antibiofilm activity and bonding performance [101]. Nanosilver-containing orthodontic cements demonstrated short-term antibacterial activity, but this effect decreased with aging and was not maintained after eight weeks [108]. ZnSO_4_-modified GIC inhibited *S. mutans* only in fresh specimens containing the highest ZnSO_4_ concentration, with no sustained antibacterial effect after 15 days [112]. Antibacterial effects were most evident for CHX-based systems, DMAHDM, MDPB, propolis, silibinin, nanosonosensitizers, nanosilver, and zinc-containing additives; however, the available data indicate that antimicrobial activity may be concentration-dependent and may diminish over time [45,96,98,101,103,105,106,108,109,112].

#### 3.3.4. Enamel Protection and Remineralization Potential

Enamel protection and remineralization-related outcomes were less frequently assessed than fluoride release or mechanical properties, but several studies provided evidence supporting the preventive potential of modified orthodontic GICs and adhesives. The most relevant approaches included the incorporation of fluoride- and calcium-releasing particles, antibacterial monomers, protein-repellent monomers, and phosphate-based additives [45,98,99,106,107,109,110].

nCaF_2_-containing orthodontic cements demonstrated enhanced fluoride release and recharge capacity, which may support long-term protection against enamel demineralization during fixed orthodontic treatment [99]. In another study, incorporation of nCaF_2_ and DMAHDM into RMGIC enhanced pH-responsive fluoride release, reduced biofilm activity, and supported remineralizing effects without impairing bond strength at optimal concentrations [106]. The combination of MPC, DMAHDM, and NACP provided superior protection against enamel prism dissolution caused by biofilm acids compared with unmodified RMGIC and resin control materials [107].

TMP-based systems also showed favorable enamel-related effects. TMP incorporation enhanced fluoride release and enamel fluoride uptake, while CHX/TMP-modified RMGICs reduced biofilm metabolism and helped inhibit enamel demineralization [109]. TMPnano combined with phosphorylated chitosan improved fluoride availability under cariogenic conditions and showed potential remineralizing activity [98]. Mineral-based additives such as BAG, CPP-ACP, hydroxyapatite, seashell-derived nanoparticles, and nanochitosan may indirectly contribute to enamel protection by increasing fluoride release, improving mechanical behavior, or reducing bacterial adhesion [42,44,45,110]. However, direct enamel demineralization or remineralization testing was not performed in all of these studies, so their protective potential should be interpreted cautiously.

#### 3.3.5. Cytotoxicity and Biological Safety

Cytotoxicity and biological safety were evaluated in a limited number of studies. Overall, the available findings suggest that selected modified glass ionomer-based materials may remain biologically acceptable when additives are incorporated at appropriate concentrations [98,99,103,106].

TMPnano/phosphorylated chitosan-modified RMGIC was reported to be non-cytotoxic, and the TMPnano + 0.25% Chi-Ph group showed favorable cell viability results [98]. nCaF_2_-containing orthodontic cements demonstrated acceptable biocompatibility, with cell viability exceeding 70% in selected groups and values comparable to commercial RMGIC controls [99]. Silibinin-modified RMGIC did not significantly reduce the viability of human gingival fibroblasts at the tested concentrations and was considered non-cytotoxic [103]. Similarly, RMGIC modified with nCaF_2_ and DMAHDM exhibited acceptable cytocompatibility, with human gingival fibroblast viability comparable to the control group at certain time points [106].

Despite these favorable findings, most included studies did not directly assess cytotoxicity, pulp-related responses, long-term biological safety, or clinical tissue compatibility. Therefore, although several additives demonstrated promising in vitro performance, biological safety should be interpreted in the context of limited experimental evidence.

To facilitate comparison across the diverse additive classes, Table 3 provides a directional summary of their reported effects on fluoride release or recharge, antibacterial or antibiofilm activity, and mechanical or physical performance relative to the corresponding control materials.

### 3.4. Quality Assessment

According to the QUIN tool, twenty of the included studies were categorized as having a medium risk of bias, with scores of 58.33% [44,51,88,96,98,100,103,105,110,112], 62.50% [104,111] or 66.67% [45,95,99,102,106,107,108,109]. The remaining three studies scored over 70% and were categorized as low risk of bias [42,97,101] (see Table 4). The methodological domains that most frequently received low scores included the lack of blinding, inadequate reporting of randomization methods, and insufficient descriptions of the personnel responsible for conducting the experimental procedures and assessing outcomes. These shortcomings were common across the included in vitro studies and significantly contributed to the predominance of studies being classified as having a medium risk of bias.

## 4. Discussion

The purpose of this systematic review was to determine the effects of functional additive incorporation on fluoride release, fluoride re-release potential, and selected biological, physical, and mechanical characteristics of orthodontic glass ionomer cements and adhesive materials. Substantial methodological heterogeneity among the included studies affected both the magnitude of the reported outcomes and their comparability [42,44,45,51,88,95,96,97,98,99,100,101,102,103,104,105,106,107,108,109,110,111,112]. As summarized in Table 1 and Table 2, the studies differed in base material composition, additive type and concentration, specimen dimensions, storage medium and pH, solution-renewal procedures, fluoride recharge protocols, analytical methods, reporting units, and observation periods [42,44,45,51,88,95,96,97,98,99,100,101,102,103,104,105,106,107,108,109,110,111,112]. These variables may influence ion diffusion and measured fluoride release independently of the effect of the incorporated additive [42,88,99,100,102,104,110,111,112]. For example, short-term measurements predominantly reflect the initial burst release, whereas longer observation periods provide more information on sustained release and recharge/re-release capacity [88,99,100,102,110,111]. Similarly, results obtained in deionized water, artificial saliva, acidic media, or pH-cycling models should not be considered directly interchangeable [88,97,100,102,107,109]. Differences in fluoride measurement techniques and data normalization further precluded reliable comparison of absolute numerical values between studies [42,45,88,100,102,104,110,111]. Consequently, the synthesis emphasized the direction, persistence, and concentration dependence of effects relative to the corresponding control within each study, rather than constructing a universal ranking of additives based on cross-study numerical comparisons. This heterogeneity precluded a meaningful meta-analysis and limits the generalizability of the findings beyond the specific materials and experimental conditions evaluated [42,44,45,51,88,95,96,97,98,99,100,101,102,103,104,105,106,107,108,109,110,111,112]. The most pronounced or most frequently reported increases in fluoride release were observed for materials modified with fluoride-, calcium-, phosphate-, apatite-, chitosan-, or selected metal nanoparticle-based additives, including nCaF_2_, TMPnano, chitosan, nanofluorapatite/nanofluorohydroxyapatite, Ag/HA/Si, hydroxyapatite, nanochitosan, SNP/ZnONP, and ZnSO_4_, CPP-ACP, and bioactive glass [42,44,45,88,97,98,99,102,104,106,109,110,111,112]. At the same time, some additives with favorable biological or antibiofilm properties did not produce a sustained or clinically meaningful increase in fluoride release, indicating that additive incorporation alone does not necessarily enhance fluoride ion availability [96,101,103]. Regarding mechanical and physical properties, several modifications improved or preserved clinically relevant material parameters [42,44,45,96,97,98,104,105,109,110,111,112]. Graphene increased microhardness and reduced surface roughness, although it also decreased radiopacity [95]. Chitosan incorporation did not adversely affect the bond strength of RMGIC [97]. Among the TMPnano/phosphorylated chitosan formulations, the combination of TMPnano with 0.25% phosphorylated chitosan showed the most favorable mechanical profile [98]. Seashell-derived nanoparticles improved compressive strength and microhardness [42], whereas nanochitosan increased compressive strength, flexural strength, and wear resistance [44]. These findings suggest that appropriate additive selection and concentration may enhance the bioactive potential of orthodontic glass ionomer-based materials while preserving, or in some cases improving, selected functional properties [42,44,45,95,97,98,104,105,109,110,111,112].

The methodological quality of the included studies also affects confidence in the overall findings. Twenty of the 23 studies were classified as having a medium risk of bias, while only three were classified as having a low risk. Although no study was categorized as having a high risk of bias, the predominance of medium-risk studies means that the reported effects should be interpreted with limited confidence. Inadequate reporting or implementation of randomization, limited blinding, and insufficient information about operators and outcome assessors may introduce selection, performance, or detection bias and may reduce the reproducibility of the experiments.

The influence of these limitations may differ according to the type of outcome [42,44,45,51,88,95,96,97,98,99,100,101,102,103,104,105,106,107,108,109,110,111,112]. Instrument-based measurements such as fluoride concentration may be less susceptible to subjective assessor judgment, but they may still be affected by sample selection, specimen allocation, calibration procedures, and incomplete reporting of experimental methods [42,45,88,100,102,104,109,110,111,112]. Mechanical, morphological, and microbiological outcomes may additionally be influenced by operator-dependent specimen preparation, testing procedures, image selection, and outcome assessment. Therefore, apparently favorable findings from individual studies, particularly those based on a single formulation or limited sample size, should not be regarded as definitive evidence of additive effectiveness [42,44,45,51,88,95,96,97,98,99,100,101,102,103,104,105,106,107,108,109,110,111,112].

Greater confidence may be placed in broad directional patterns that were reproduced across more than one study or outcome domain, such as the concentration-dependent balance between enhanced bioactivity and deterioration of selected mechanical properties [99,101,102,103,108,109,111,112]. Nevertheless, because most formulations were evaluated in single, methodologically heterogeneous studies with a medium risk of bias, the present synthesis should be considered hypothesis-generating rather than confirmatory [42,44,45,51,88,95,96,97,98,99,100,101,102,103,104,105,106,107,108,109,110,111,112]. These limitations reinforce the need for independently replicated studies using prespecified sample-size calculations, appropriate randomization, blinded or automated outcome assessment where feasible, and complete reporting of operators, procedures, and analytical methods [42,44,45,51,88,95,96,97,98,99,100,101,102,103,104,105,106,107,108,109,110,111,112]. The most pronounced antibacterial and antibiofilm effects were observed for materials containing additives with targeted antimicrobial mechanisms, such as CHX, DMAHDM, MDPB, propolis, silibinin, nanosonosensitizers, nanosilver, and zinc-containing compounds [45,96,101,103,105,106,108,109,112]. Formulations combining more than one preventive mechanism appear particularly relevant, as demonstrated by the nCaF_2_/DMAHDM-modified RMGIC [106]. This combination markedly reduced biofilm viability, metabolic activity, polysaccharide production, lactic acid production, and colony-forming unit counts. In addition, it maintained a favorable fluoride-release profile and acceptable cytocompatibility [106]. A key limitation of the observed antibacterial activity was its dependence on additive concentration, exposure time, and durability after material aging [96,101,103,105,108,109,112]. Chlorhexidine-modified halloysite nanotubes inhibited *S. mutans*, particularly at higher concentrations; however, the long-term persistence of this antibacterial effect was not clearly established [105]. Nanosilver-containing cements showed reduced antibacterial activity with aging and no sustained effect after eight weeks [108], whereas ZnSO_4_ inhibited *S. mutans* only in fresh specimens containing the highest additive concentration [112]. Similarly, CHX/TMP systems improved antibiofilm activity, but higher additive concentrations compromised selected mechanical properties [109]. Therefore, in vitro antibacterial activity alone is insufficient to determine clinical usefulness; long-term antibiofilm durability, aging resistance, fluoride release, and mechanical stability should be considered together [96,101,103,105,108,109,112]. These observations are consistent with the broader literature indicating that glass ionomer cements remain valuable because of their fluoride release and chemical adhesion. However, their modification to enhance bioactivity requires careful optimization, as improving ion release or antibacterial activity may occur at the expense of mechanical properties, surface stability, or the setting process [11,12,15,19,32,33,38,39,40,41,43,45,57,96,101,103,105,108,109,112]. This concept is consistent with current trends in biomaterials research, where advanced nanostructured systems are increasingly being engineered to combine multiple biological functions while maintaining material stability and clinical applicability [99,101,104,106,107,108,111,113]. In orthodontics, this issue is particularly important because white spot lesions remain a common complication of fixed appliance treatment [106,107,109]. Therefore, the clinical value of fluoride-releasing or nanoparticle-modified materials depends not only on their initial bioactivity, but also on the durability of their effect, material stability, and confirmation of efficacy in standardized clinical trials [7,8,9,14,99,101,106,107,108,109,114,115].

Fluoride release depends on several factors, including environmental pH, the powder-to-liquid ratio, temperature, storage medium, and, most importantly, the chemical composition of the glass ionomer cement [11,15,19,20,42,47,70,88,99,100,102,109,110,111,112,114,115,116,117,118]. The methodology used for fluoride-release assessment also varied considerably among the included studies. Fluoride ion-selective electrodes were the predominant analytical method and were used in 11 studies [42,88,99,101,103,104,106,109,110,111,112]. Considerable heterogeneity was also observed with respect to storage media, including distilled water, deionized water, artificial saliva with different compositions and pH values, saline solution, lactic acid solution, and pH-cycling models simulating alternating demineralization and remineralization conditions [88,97,100,102,107,109]. The duration of fluoride-release assessment ranged from 24 h to six months, with sampling intervals varying from hours to weekly or monthly measurements [45,88,98,100,102,104,109,110,111,112]. Only a limited number of studies extended fluoride-release testing to include fluoride recharge and/or re-release assessment, particularly for materials modified with nano-calcium fluoride, nanofluorapatite, silver–hydroxyapatite–silica nanoparticles, silibinin, or zinc-containing additives [88,99,103,104,112]. These methodological differences are likely to influence both the magnitude and kinetics of fluoride release, as acidic storage media generally promote greater fluoride release than neutral conditions, whereas longer observation periods enable sustained fluoride release and recharge/re-release phenomena to be evaluated [88,99,100,102,109,112]. Consequently, direct quantitative comparison of fluoride-release values between studies should be interpreted with caution, and greater emphasis should be placed on overall trends rather than absolute fluoride-release values [42,44,45,51,88,95,97,98,99,100,101,102,103,104,105,106,107,108,109,110,111,112]. The present analysis showed that glass ionomer cements incorporating nano-calcium fluoride generally exhibited increased fluoride release, with this effect being particularly evident under acidic conditions [99,106]. In two studies, this increase was attributed to the smaller particle size of nCaF_2_ compared with conventional glass particles, which may increase the available surface area and facilitate fluoride ion diffusion from the material [99,106]. Other fluoride-reservoir additives associated with enhanced fluoride release included nanofluorapatite [88,102] and nanofluorohydroxyapatite [102]. Enhanced fluoride release was also reported after incorporation of TMPnano, which may be related to its nanoscale particle size and high surface area [98]. More broadly, materials-science research indicates that nanostructures such as quantum dots and MXenes may exhibit enhanced surface-dependent reactivity owing to their high specific surface area and abundance of exposed active sites; however, evidence from electrocatalytic systems provides only general physicochemical context and cannot be directly extrapolated to dental glass ionomer cements [116]. In addition, the affinity of TMP for calcium and hydroxyapatite may promote mineral interactions that increase fluoride availability in the surrounding environment [98,109]. Chitosan and nanochitosan were also associated with enhanced fluoride release from GICs [44,97]. Patel et al. observed significantly higher fluoride release from chitosan-modified RMGIC after 15 and 30 days [97], whereas Kumar et al. reported increased fluoride release from nanochitosan-modified GIC at all evaluated time points [44]. The proposed explanation is that chitosan may facilitate fluoride diffusion through the cement matrix, while nanochitosan may additionally promote early aluminum polyacrylate formation and provide a larger reactive surface area, thereby further supporting fluoride release [44,97].

Although only a limited number of studies evaluated fluoride recharge and/or re-release, the available evidence indicates that nano-calcium fluoride and nanofluorapatite are the most promising additives for enhancing long-term fluoride replenishment [88,99]. Nano-calcium fluoride demonstrated sustained fluoride re-release over multiple recharge cycles during a 70-day observation period, whereas nanofluorapatite demonstrated enhanced fluoride recharge throughout an 84-day evaluation period, with higher nanofluorapatite concentrations resulting in greater recharge capacity [88,99]. More limited evidence is available for materials modified with silver–hydroxyapatite–silica nanoparticles, silibinin, and zinc-containing additives, which were also evaluated for fluoride recharge and/or re-release, although these findings are currently based on individual studies [88,103,104,112]. These findings suggest that fluoride replenishment may extend beyond the initial days or weeks; however, additional standardized long-term studies are required to confirm the durability and clinical relevance of this effect [88,99,103,104,112]. The findings related to antibacterial activity, enamel protection against demineralization, and biological safety further support the potential clinical value of modified orthodontic glass ionomer materials [45,96,98,101,103,105,106,107,108,109,112]. Several additives, including chlorhexidine-modified halloysite nanotubes, CHX/TMP systems, DMAHDM, MDPB, red propolis, silibinin, ultrasound-activated nanosonosensitizers, nanosilver-containing cements, and zinc-containing compounds, demonstrated antibacterial or antibiofilm activity against cariogenic microorganisms such as *Streptococcus mutans*, *Candida albicans*, and/or *Lactobacillus acidophilus* [45,96,101,103,105,106,108,109,112]. Enamel-protective and remineralization-related effects were reported mainly for formulations containing nCaF_2_, DMAHDM, MPC, NACP, TMP, and CHX/TMP systems [98,99,106,107,109]. Other additives, such as hydroxyapatite, CPP-ACP, bioactive glass, and chitosan-based systems, may indirectly contribute to enamel protection by enhancing fluoride or ion release, improving material properties, or reducing bacterial adhesion; however, direct enamel demineralization or remineralization testing was not performed in all of these studies [44,45,97,98,110]. Available evidence also suggests acceptable biocompatibility of selected modified materials, including formulations containing TMPnano, phosphorylated chitosan, nCaF_2_, DMAHDM, and silibinin [98,99,103,106]. Collectively, these findings indicate that contemporary modifications may provide multifunctional benefits by combining fluoride release with antibacterial and potential enamel-protective properties [45,96,98,101,103,105,106,107,108,109,112].

Although increased fluoride release is generally considered beneficial for reducing enamel demineralization and promoting remineralization [106,107,109], the currently available evidence does not allow the definition of a universal clinically effective fluoride-release threshold [42,44,45,51,88,95,96,97,98,99,100,101,102,103,104,105,106,107,108,109,110,111,112]. Furthermore, the clinical balance between demineralization and remineralization depends on numerous patient- and environment-related factors, including salivary flow and composition, oral pH, plaque accumulation, dietary habits, oral hygiene, and individual caries risk [117]. Therefore, increased fluoride release should be interpreted as an indicator of greater preventive potential rather than definitive evidence of clinical effectiveness [42,44,45,51,88,95,96,97,98,99,100,101,102,103,104,105,106,107,108,109,110,111,112].

From a clinical perspective, the applicability of additive-modified orthodontic glass ionomer materials depends on the intended clinical objective. Fluoride-containing additives, particularly nano-calcium fluoride, nanofluorapatite, nanofluorohydroxyapatite, and TMPnano, appear to be the most suitable choice for patients at high risk of enamel demineralization because they consistently enhanced fluoride release and, in some studies, fluoride recharge/re-release [88,98,99,102,106,109]. In contrast, antimicrobial additives such as DMAHDM, MDPB, chlorhexidine, silibinin, and nanosilver may be advantageous in situations where biofilm control is the primary therapeutic objective, although some formulations were associated with reduced mechanical properties or limited long-term antibacterial durability [45,103,105,106,107,108,109]. Bioactive additives, including chitosan, nanochitosan, hydroxyapatite, CPP-ACP, bioactive glass, and seashell-derived nanoparticles, generally provided a more balanced combination of mechanical reinforcement and preventive properties, making them attractive candidates for routine clinical use [42,44,45,97,98,109]. These findings suggest that the clinical applicability of additive-modified orthodontic glass ionomer materials should be based on the patient’s individual caries risk and treatment priorities, rather than on maximizing a single material property [42,44,45,88,97,98,99,100,101,102,103,104,105,106,107,108,109,110].

From a clinical perspective, the in vitro findings suggest that selected additive-modified formulations may have potential as adjunctive materials for orthodontic patients at increased risk of enamel demineralization, particularly when sustained fluoride release or recharge capacity and antibiofilm activity are achieved without substantial deterioration of mechanical performance or cytocompatibility [98,99,101,103,106,107,108,109]. However, these laboratory findings do not establish clinical effectiveness or suitability for routine use [42,44,45,51,88,95,96,97,98,99,100,101,102,103,104,105,106,107,108,109,110,111,112]. Intraoral performance is influenced by saliva and acquired pellicle formation, multispecies biofilms, fluctuating pH, dietary challenges, exposure to fluoride-containing oral hygiene products, moisture contamination, thermal changes, and repeated mechanical loading throughout orthodontic treatment. These factors may alter fluoride diffusion, reduce the durability of antibacterial activity, and influence material degradation, wear, marginal integrity, and bracket or band retention [88,97,99,100,101,102,106,107,108,109]. Furthermore, short-term cytocompatibility findings do not exclude chronic tissue responses, the release of unreacted components or nanoparticles, or adverse effects associated with prolonged intraoral exposure [98,99,103,106]. Before clinical implementation, modified materials should demonstrate reproducible handling and setting characteristics, long-term physicochemical stability, durable fluoride and antibiofilm effects, acceptable bracket or band failure rates, and biological safety under clinically relevant aging and multispecies biofilm conditions [98,99,101,103,106,107,108,109]. Prospective clinical studies should subsequently evaluate outcomes such as the incidence and severity of white spot lesions, bracket or band failure, enamel damage during debonding, gingival or mucosal adverse reactions, and material durability over clinically relevant treatment periods [99,106,107,108,109]. Until such evidence is available, these formulations should be regarded as experimental and should not replace established oral hygiene and caries-prevention measures [42,44,45,51,88,95,96,97,98,99,100,101,102,103,104,105,106,107,108,109,110,111,112].

Several limitations of this review should be acknowledged. First, the topic remains relatively underexplored, and many additives have been evaluated in only a small number of studies, which limits the strength of the available evidence [42,44,45,51,88,95,96,97,98,99,100,101,102,103,104,105,106,107,108,109,110,111,112]. Second, only English-language publications were included, which may have introduced language bias. The methodological heterogeneity described above limits the certainty and transferability of the narrative synthesis [42,44,45,51,88,95,96,97,98,99,100,101,102,103,104,105,106,107,108,109,110,111,112]. Accordingly, the reported findings should be interpreted primarily as study-specific comparative evidence rather than as a basis for universal rankings, pooled effect estimates, or quantitative clinical thresholds [42,44,45,51,88,95,96,97,98,99,100,101,102,103,104,105,106,107,108,109,110,111,112]. Future investigations should adopt a predefined minimum set of experimental and reporting parameters. The base material should be identified by product name, manufacturer, batch, material category, powder-to-liquid ratio or resin composition, mixing procedure, and curing conditions [42,44,45,51,88,95,96,97,98,99,100,101,102,103,104,105,106,107,108,109,110,111,112]. Additives should be characterized by their chemical composition, particle size and size distribution, morphology, surface functionalization, dispersion method, and incorporated concentration [42,44,45,51,88,95,98,99,101,102,103,104,105,106,107,108,109,110,111,112]. The basis used to calculate concentration should be explicitly stated, for example as a percentage of the powder mass, liquid mass, or total mixed material [42,44,45,51,88,95,96,97,98,99,100,101,102,103,104,105,106,107,108,109,110,111,112]. Studies should include an unmodified control and, where appropriate, a commercially available reference material [42,44,45,51,88,95,96,97,98,99,100,101,102,103,104,105,106,107,108,109,110,111,112]. Concentration-response experiments should preferably evaluate several prespecified additive levels rather than a single arbitrary concentration [42,44,45,51,88,95,96,97,98,99,102,104,105,106,109,110,111,112].

Fluoride-release studies should standardize or fully report the analytical method, instrument calibration, use of ionic-strength adjustment buffers, specimen dimensions and exposed surface area, number of replicates, storage-medium composition and volume, pH, temperature, agitation, and solution-renewal schedule [42,44,45,51,88,95,97,98,99,100,102,103,104,106,109,110,111,112]. Ion-selective electrode measurements and ion chromatography may both be appropriate, if validation and calibration procedures are clearly described [42,88,99,100,103,104,106,109,110,111,112]. Results should distinguish interval from cumulative fluoride release and should be expressed in comparable normalized units, preferably relative to specimen mass or exposed surface area [42,44,45,51,72,76,77,88,95,99,100,109,110,111,112,118,119,120,121].

A common minimum set of observation points should capture both initial burst release and sustained release [45,88,99,100,102,104,110,112]. Suggested core time points include 24 h, 7 days, 28 days, and at least one longer-term assessment at three months or later, reflecting the wide range of observation periods used in the available literature [42,44,51,88,95,98,99,100,101,102,103,104,109,110,111,112,119]. Fluoride recharge and re-release experiments should additionally standardize the fluoride source and concentration, duration and method of application, rinsing procedure, number of recharge cycles, and post-recharge sampling intervals [88,99,103,104,112,121].

Storage conditions should include a clearly defined neutral reference environment at 37 °C [100]. When artificial saliva is used, its composition should be reported in full [97,102]. Acidic media, pH-cycling protocols, or demineralization–remineralization models should be investigated as separate challenge conditions and should not be treated as directly interchangeable with neutral storage [88,107,109]. Aging procedures should specify thermocycling parameters, water-storage duration, mechanical loading, and any simulated dietary or fluoride exposures [72,76,77,86,88,91,99,101,102,108,109,118,120,121].

For mechanical testing, future studies should standardize specimen geometry, tooth substrate, enamel-conditioning procedure, bracket-base type and area, adhesive thickness, crosshead speed, loading configuration, storage and aging conditions, and failure-mode assessment [96,98,99,101,102,103,106,107,108,109]. Antibacterial studies should report microorganism strains, inoculum density, biofilm composition, incubation period, growth medium, aging of the material before testing, and the method used to quantify antibacterial or antibiofilm activity [45,96,98,101,103,105,106,108,109,112]. Cytocompatibility studies should specify the tested cell type, extraction ratio, exposure duration, dilution, and viability-assessment method [98,99,103,106]. Across all experimental domains, prespecified sample-size calculations, random allocation, independent or automated outcome assessment where feasible, and complete reporting of raw and normalized results would improve reproducibility and facilitate future quantitative synthesis [42,44,45,51,87,88,95,96,97,98,99,100,101,102,103,104,105,106,107,108,109,110,111,112]. Finally, all included studies were conducted in vitro. Therefore, the review can identify promising material profiles and comparative laboratory trends but cannot determine clinical effectiveness, long-term intraoral durability, patient safety, or treatment-related failure rates [42,44,45,51,88,95,96,97,98,99,100,101,102,103,104,105,106,107,108,109,110,111,112]. Further research should progress from standardized aging, pH-cycling, fluoride recharge, and multispecies biofilm models to well-designed prospective clinical studies and randomized controlled trials [88,99,101,103,106,107,108,109,112]. An additional limitation concerns the interpretation of shear bond strength data. The included studies used heterogeneous protocols, including different tooth substrates and surface-conditioning procedures, bracket-base designs and areas, storage conditions, aging or thermocycling procedures, crosshead speeds, loading configurations, and specimen geometries [96,97,99,101,102,106,108]. These factors can substantially influence measured SBS values. Therefore, absolute values from different studies were not considered directly interchangeable, and no universal SBS threshold was used to establish clinical acceptability [96,97,99,101,102,106,108]. The findings were interpreted primarily through within-study comparisons between modified formulations and their corresponding controls [96,97,99,101,102,106,108]. In vitro SBS results should consequently be regarded as indicators of comparative mechanical behavior rather than proof of clinical performance or suitability [96,97,99,101,102,106,108].

## 5. Conclusions

The findings of this systematic review suggest that the incorporation of functional additives into orthodontic glass ionomer cements and glass ionomer-based adhesives may enhance their preventive potential, particularly by improving fluoride release, fluoride recharge/re-release capacity, antibacterial activity, and potential enamel-protective effects. Among the investigated additive systems, nCaF_2_-containing formulations—particularly those combining nCaF_2_ with DMAHDM—showed one of the most promising multidomain profiles, combining sustained or rechargeable fluoride release with antibacterial and remineralizing effects while preserving bond strength and cytocompatibility at optimized concentrations [99,106]. TMPnano combined with phosphorylated chitosan [98], CHX/TMP formulations [109], and the MPC/DMAHDM/NACP system [107] also demonstrated favorable combinations of fluoride-related, antibacterial, enamel-protective, and mechanical outcomes under the respective experimental conditions. However, these systems should be regarded as candidates for further investigation rather than as a definitive ranking, because most formulations were evaluated in single, methodologically heterogeneous in vitro studies and overall confidence in the evidence remains limited. Consequently, no modified formulation can currently be recommended for routine orthodontic use. Standardized long-term laboratory studies should be followed by adequately powered, long-term randomized controlled clinical trials assessing white spot lesion development, bracket or band failure, material durability, enamel damage, and adverse tissue reactions before definitive clinical recommendations can be established.

## Figures and Tables

**Table 1:** General characteristics of studies.

Study	Aim of the Study	Materials and Methods	Results	Conclusions
Jordão et al. [95]	Evaluation of different properties of graphene-reinforced GICs (mechanical, physical, chemical, biological).	Two self-curing GICs (Ketac Molar easy mix (3M ESPE, Monrovia, CA, USA), Fuji IX(GC Corp, Tokyo, Japan)) were mixed with graphene powder resulting at different concentrations (0% [graphene-free control group]; 0.5%, 1%, 2%, and 5%). After the testing process all samples were assessed by color, surface roughness evaluation by non-contact profilometry, radiopacity, sorption and solubility, microhardness, antibacterial properties and fluoride release.	The control groups exhibited increased volumetric (Sa) and linear (Ra) roughness values. No antibacterial properties were observed. The incorporation of graphene decreased radiopacity. After 21 days, solubility and water sorption did not differ between the evaluated groups. Higher fluoride release was observed after 7 days in most groups and after 21 days in the 1%, 2%, and 5% Ketac groups.	Incorporation of graphene into GICs at low concentrations, particularly 0.5% and 1%, enhanced microhardness without affecting the evaluated biological properties.
de Morais Sampaio et al. [96]	Assessment of antimicrobial, mechanical properties and fluoride release of GIC (used for cementing orthodontic bands) modified by addition of extract of red propolis (EERP) at different concentrations	Two orthodontic GICs were mixed with red propolis (EERP) at different concentrations (10%, 25%, and 50%) and tested for antimicrobial activity, selected mechanical properties, such as diametral tensile strength (DTS), shear bond strength (SBS) and compressive strength (CS), microhardness and fluoride release. Two-way ANOVA followed by Tukey’s test was used for antimicrobial tests, fluoride release, DTS, CS, and microhardness (*p* < 0.05). One-way ANOVA with Tukey’s test was applied (*p* < 0.05) to analyze shear bond strength	Addition of EERP reduced the viability of *S. mutans* and *C. albicans*, with stronger effects at higher concentrations (notably 25% and 50%). Meron showed higher DTS, but adding 10% and 50% propolis weakened DTS; CS was mostly unchanged, except 50% was lower than 25%. No significant differences were found between materials or propolis concentrations in microhardness or fluoride ion release. Riva showed higher shear bond strength than Meron, while propolis concentration did not significantly affect bonding.	The 25% EERP GIC group showed a substantial increase in antimicrobial activity against *S. mutans* and *C. albicans*, without significant changes in fluoride release or mechanical properties.
Patel et al. [97]	The study aimed to evaluate adhesive bond strength and sustained fluoride release in chitosan-infused RMGIC.	Twenty extracted premolars were randomly divided into two groups: - I standard RMGIC - II chitosan-modified RMGIC. Teeth were stored in artificial saliva for 30 days and kept under controlled conditions. Fluoride release was measured on days 15 and 30. Adhesive bond strength was tested using a universal testing machine.	The mean adhesive bond strength did not differ significantly between the two study groups. Fluoride release: Group I: fluoride release was lower at day 15 than at day 30. Group II: fluoride release was lower at day 15 than at day 30. At day 15, mean fluoride release was significantly higher in Group II than in Group I. At day 30, mean fluoride release was markedly higher in Group II than in Group I. The mean percentage change in fluoride release by day 30 did not differ significantly between groups.	Chitosan-infused RMGIC preserved adhesive bond strength and increased fluoride release relative to unmodified RMGIC under the tested conditions.
Peres Fernandes et al. [98]	This study evaluated the mechanical, antimicrobial, and cytotoxic properties and fluoride release of RMGIC containing TMPnano and phosphorylated chitosan (Chi-Ph).	RMGIC was modified with Chi-Ph (0.25% or 0.5%) and/or TMPnano (14%), and the following properties were assessed: - diametral compressive and tensile strength (DCS/TS), surface hardness (SH), and degree of conversion (%DC); - fluoride release during demineralization/remineralization cycles; - antimicrobial/antibiofilm activity (agar diffusion and XTT biofilm-metabolism assays); - cytotoxicity in fibroblasts (resazurin assay).	Phosphorylated chitosan (Chi-Ph) was successfully obtained (~75% yield). The combination of 14% TMPnano with 0.25% Chi-Ph showed the best mechanical properties without affecting the degree of conversion. All groups showed high initial fluoride release that decreased over time; TMPnano-containing groups showed the highest release. The combination of TMPnano and 0.25% Chi-Ph had the best antibacterial and antibiofilm activity against *S. mutans*. All materials were non-cytotoxic at 24 h (the TMPnano + 0.25% Chi-Ph group showed the lowest cytotoxicity after 72 h).	RMGIC containing 14% TMPnano and 0.25% Chi-Ph preserved physicomechanical properties, was non-cytotoxic to fibroblasts, and reduced *S. mutans* viability and metabolism.
Albasso et al. [42]	To evaluate the influence of incorporating seashell-derived nanoparticles into glass-ionomer cement on its mechanical properties and fluoride ion release.	Conventional glass-ionomer cement (Medifil, PROMEDICA Dental Material GmbH, Germany) was used as the base material. Seashell-derived nanoparticles were obtained through mechanical grinding and subsequently characterized by TEM and EDX analysis. The glass ionomer cement was modified by incorporating nanoparticles at concentrations of 5 wt% and 10 wt%, and the specimens were allocated into three groups: control, 5%, and 10% (*n* = 75). The specimens were examined for surface microhardness, compressive strength and fluoride ion release. After 1 and 4 weeks using an ion-selective electrode. FTIR analysis was performed to assess structural changes.	Modification with seashell nanoparticles significantly improved compressive strength and microhardness, with the highest values observed in the 10% group. Fluoride release was enhanced in both modified groups compared with the control, particularly after 4 weeks, with the 10% group showing the highest values. No notable differences in chemical structure were detected between groups based on FTIR analysis. All groups demonstrated increased fluoride release over time.	The incorporation of seashell nanoparticles, especially at 10%, improves the mechanical performance of glass-ionomer cement and enhances its fluoride-releasing ability without altering its chemical structure.
Yi et al. [99]	To develop a novel orthodontic cement containing nano-calcium fluoride (nCaF_2_) with enhanced long-term fluoride release and recharge capability, and to evaluate its mechanical properties and biocompatibility.	Experimental orthodontic cements were formulated using resin matrices (PMGDM and EBPADMA, with or without HEMA and BisGMA) and incorporated with nano-CaF_2_ particles (20 wt% and 30 wt%). A commercial resin-modified glass ionomer (RMGIC) served as control. Nanoparticles were synthesized via spray-drying and characterized by TEM. Samples were evaluated for shear bond strength to enamel (before and after demineralization challenge), fluoride release over 70 days, fluoride recharge and re-release over multiple cycles, and cytotoxicity using human gingival fibroblasts.	The experimental cements showed bond strength comparable to the commercial control and maintained performance after demineralization. Materials containing nCaF_2_ demonstrated significantly higher and more sustained fluoride release compared with RMGIC. After fluoride recharge, experimental groups exhibited prolonged re-release, while the control showed only short-term release. Increasing nanoparticle content (30%) enhanced both fluoride release and recharge capacity. Cytotoxicity remained within acceptable limits.	The developed nCaF_2_-containing orthodontic cement provides sustained and rechargeable fluoride release while maintaining adequate mechanical properties and biocompatibility, suggesting its potential for long-term prevention of enamel demineralization during orthodontic treatment.
Li et al. [100]	To develop and evaluate a modified orthodontic bracket capable of releasing fluoride by incorporating fluoride-containing materials into its structure.	Metal orthodontic brackets were modified by creating a recess in the base, which was filled with different materials: resin-modified glass ionomer cement (RMGIC), glass ionomer cement (GIC), zinc phosphate cement, and composite resin, with or without added sodium fluoride. A total of 32 brackets were divided into groups depending on material type and fluoride addition. After storage in deionized water at 37 °C, fluoride release from the samples was determined using ion chromatography, with measurements performed daily for the first 7 days and weekly thereafter up to 10 weeks.	All fluoride-containing groups showed an initial high fluoride release followed by a rapid decrease. The composite with high sodium fluoride content exhibited the highest release in the first two weeks but declined sharply thereafter. RMGIC demonstrated a more stable and sustained fluoride release over time compared with other materials. Materials without fluoride addition showed minimal or no release.	Incorporating fluoride-releasing materials into modified orthodontic brackets can provide sustained fluoride delivery. Among the tested materials, RMGIC offered the most consistent long-term fluoride release, suggesting its suitability as a fluoride reservoir in orthodontic applications.
Pourhajibagher et al. [101]	To evaluate the physicomechanical properties, fluoride release, and antibiofilm activity of resin-modified glass ionomer containing ultrasound-activated nanosonosensitizers against *Streptococcus mutans*.	Resin-modified glass ionomer (GC Fuji Ortho LC) was modified with nano-curcumin (n-Cur), nano-emodin (n-Emo), and nano-quercetin (n-Qct) at concentrations of 2%, 5%, and 10% (wt%). A total of 50 human molars were used. Samples were tested for shear bond strength (SBS), adhesive remnant index (ARI), setting time, and fluoride release (measured at 1, 7, 15, and 30 days using an ion-selective method). Antibiofilm activity against *S. mutans* was assessed after ultrasound activation by CFU counting and FESEM analysis.	Modification of glass ionomer reduced SBS in a dose-dependent manner. The 5% n-Emo, 5% n-Cur, and 2% n-Qct formulations showed less pronounced SBS reductions than the corresponding higher-concentration formulations. ARI scores did not differ significantly between groups. Fluoride release increased over time in all groups and was not significantly affected by nanoparticle incorporation. Setting time was reduced in modified materials, especially with n-Emo. Ultrasound-activated groups showed a significant reduction in *S. mutans* biofilm, with the strongest antibacterial effect observed for 5% n-Emo.	Incorporation of nanosonosensitizers into resin-modified glass ionomer enhances antibacterial properties without compromising fluoride release. The 5% nano-emodin formulation showed the most favorable balance between mechanical performance and antimicrobial activity among the tested formulations under the experimental conditions used.
Lin et al. [102]	The study aimed to evaluate the influence of nano-fluorohydroxyapatite (FHA) and nano-fluorapatite (FA) incorporation into a resin-modified glass ionomer cement on fluoride ion release and bond strength to enamel	In the present study, nano-FA and nano-FHA were incorporated into resin-modified glass ionomer cement (Fuji Ortho LC) at concentrations ranging from 2.5 wt% to 30 wt%. Specimens were prepared and stored in artificial saliva. Fluoride release was measured over 70 days using spectrophotometry. Based on preliminary results, 25% concentration was selected for further testing. Shear bond strength (SBS) and micro-tensile bond strength (µTBS) were evaluated using human enamel samples. SEM and XRD analyses were also performed.	Incorporation of nano-FA and nano-FHA increased fluoride release, with the highest values observed at 25%, particularly for the FA group. A typical initial burst release was followed by a decrease and then a gradual increase over time. Modified materials showed reduced SBS and µTBS compared with the control, especially in the FHA group. Nano-FA showed a more favorable balance between fluoride release and bond strength than nano-FHA under the experimental conditions used.	The addition of fluoride-containing apatite nanoparticles enhances the fluoride-releasing capacity of resin-modified glass ionomer cement. Nano-FA provided a more favorable balance between fluoride release and mechanical performance compared with nano-FHA.
Parizi et al. [103]	This study assessed the bacteriostatic activity of silibinin-modified light-cured RMGIC used in orthodontics (GC Fuji Ortho LC, GC Corporation, Tokyo, Japan). Material properties, including fluoride release, were also evaluated.	A total of 45 samples were used. Silibinin at concentrations of 0%, 1%, 2%, and 5% was incorporated into the liquid component of the bracket-bonding adhesive and mixed with the powder according to the manufacturer’s recommended ratio. Samples were tested for fluoride release, antibacterial activity, shear bond strength, and cytotoxicity.	The tests showed that: - samples containing 5% silibinin showed a stronger bacteriostatic effect than samples containing lower concentrations. - fluoride release was initially similar across groups; samples containing silibinin showed slightly greater recharge on subsequent days, but no substantial differences remained after 30 days. - silibinin caused a concentration-dependent reduction in shear bond strength, with greater reductions at higher concentrations. - none of the tested groups exhibited cytotoxic effects.	In conclusion, silibinin enhanced the antibacterial activity of RMGIC but did not substantially affect fluoride release or recharge.
Dobrzynski et al. [88]	The study aimed to compare fluoride release from glass ionomer cements containing nanofluorapatite with that from GC Fuji Ortho RMGIC under different ionic and pH conditions.	GC Fuji Ortho RMGIC containing 0%, 2%, or 5% nanofluorapatite was evaluated in nine media with different ionic compositions and pH values (five samples per condition; 135 specimens in total). Fluoride recharge was tested repeatedly over 84 days.	All groups showed their highest fluoride release between the first and third hours. Fluoride recharge in samples containing 5% nFAp was substantially higher than in the other groups.	Nanofluorapatite altered the cement texture and increased fluoride release. Higher nFAp concentrations produced greater fluoride recharge.
Raghimi et al. [104]	The aim of the study was to modify resin-modified glass ionomer cement (Fuji II LC) with silver–hydroxyapatite–silica hybrid nanoparticles and assess the impact of this modification on the cement’s fluoride release capability and compressive strength.	Sixty disk specimens measuring 4 mm in diameter and 6 mm in height were fabricated and divided into six groups (*n* = 10): pure GC Fuji II LC resin-modified glass ionomer, BracePaste composite, and GC Fuji II LC reinforced with Ag/HA/Si hybrid nanoparticles at concentrations of 0.1 wt%, 0.5 wt%, 1 wt%, and 2 wt%. The specimens were submitted to a compressive strength test. This test was conducted using a universal testing machine. Fluoride content and fluoride-releasing capacity were additionally assessed using a digital ion-selective electrode in disk-shaped specimens measuring 3 mm in diameter and 2 mm in height. These disks were prepared from unmodified GC Fuji II LC RMGIC and from RMGIC reinforced with Ag/HA/Si hybrid nanoparticles at concentrations of 0.1 wt%, 0.5 wt%, 1 wt%, and 2 wt%.	A compressive strength test established that BracePaste had the highest resistance to pressure, whereas the glass ionomer cements showed lower resistance, with no significant differences among the tested materials. The fluoride recharge was substantially higher in cements with incorporated Ag/HA/Si hybrid nanoparticles in comparison to the control sample.	In conclusion, incorporation of Ag/HA/Si hybrid nanoparticles did not substantially affect material strength, but higher concentrations increased fluoride release.
Salmerón-Valdés et al. [105]	To evaluate the antibacterial and mechanical properties of type I glass ionomers reinforced with 2% chlorhexidine-modified halloysite nanotubes at 5% and 10% wt.	A total of 200 samples were used for antibacterial testing and divided into four experimental and six control groups (20 each). Mechanical properties were evaluated in 270 samples for microhardness, compressive strength, and setting time. Group characterization was performed by SEM and FTIR, and antibacterial activity against *Streptococcus mutans* was assessed after 24 h	No antibacterial effect was observed in the control or positive control groups. The 5% and 10% experimental groups showed inhibition zones of 11.35–11.45 mm and 12.50–13.20 mm, respectively, with a significant difference between them. Microhardness and setting time did not differ significantly from controls, whereas compressive strength was significantly higher, particularly in the 10% nanotube group.	The addition of nanotubes at 5% and 10% effectively reduced the presence of *S. mutans*, showing a dose-dependent effect, while preserving and enhancing the mechanical properties of the material.
Yi et al. [106]	To develop and assess a novel resin-modified glass ionomer (RMGIC) with nCaF_2_ and dimethylaminohexadecyl methacrylate (DMAHDM) for antibacterial and remineralizing effects.	20 wt% nCaF_2_ and 3 wt% DMAHDM were incorporated into a RMGIC (GC Ortho LC). The modified cement was evaluated for bond strength, cytotoxicity, ion release, antibacterial activity, and remineralization potential.	The addition of 20 wt% nCaF_2_ and 3 wt% DMAHDM maintained bond strength, enhanced pH-dependent ion release, reduced biofilm activity and CFU counts, improved enamel remineralization, and showed acceptable cell viability.	The nCaF_2_/DMAHDM-modified cement improved antibacterial activity, remineralization, and enamel hardness without adversely affecting bracket bond strength or biocompatibility.
Ma et al. [107]	Development and in vitro evaluation of a bioactive multifunctional orthodontic cement with protein-repellent, antibacterial, and remineralizing effects.	2-methacryloyloxyethyl phosphorylcholine (MPC), dimethylaminohexadecyl methacrylate (DMAHDM), and nanoparticles of amorphous calcium phosphate (NACP) were incorporated into a resin-modified glass ionomer (RMGIC). Extracted human teeth with bonded brackets were assigned to 4 material groups. Enamel demineralization was evaluated using a microcosm biofilm model. Lesion depth and enamel hardness were evaluated using polarized light microscopy (PLM) and hardness testing, respectively.	The addition of MPC, DMAHDM, and NACP did not impair bond strength. The lowest lesion depth in enamel was observed in the RMGIC+MPC+DMAHDM+NACP group. The highest enamel hardness was observed in the NACP-containing groups. RMGIC, MPC and DMAHDM exhibited a more pronounced effect on demineralization inhibition than RMGIC and TB controls. RMGIC, MPC, DMAHDM and NACP exhibited superior efficacy in protecting enamel prisms from dissolution by biofilm acids compared with RMGIC and TB control groups.	The novel RMGIC+MPC+DMAHDM+NACP cement reduced enamel demineralization around brackets more effectively than commercial controls and showed promising anti-caries potential.
Li et al. [108]	Shear bond strength and long-term antibacterial activity were evaluated in experimental cements modified with nanosilver.	Experimental nanosilver-containing glass ionomer cements (NSC) prepared at five concentrations. The antibacterial activity of three orthodontic cement products and five nanosilver-containing cement (NSC) samples was assessed using the direct contact test (DCT) and agar diffusion test (ADT), with DCT results based on turbidity measurements. Bacterial growth in the DCT was assessed by turbidity measurements in 96-well microtiter plates following aging of the tested materials for 1 day and for 1, 2, 3, 4, 6, and 8 weeks. Shear bond strength of 3 orthodontic cements and 5 NSC formulations was evaluated with a universal testing machine.	Fresh NSC samples showed strong antibacterial activity, but this effect decreased with aging and was absent after 8 weeks. Increasing nanosilver content reduced bond strength, and the 15% nanosilver formulation showed significantly lower SBS than the control.	NSCs may reduce enamel demineralization around brackets without adversely affecting bond strength.
da Silva et al. [109]	To evaluate the effect of CHX and nano-sized sodium trimetaphosphate incorporation into RMGIC on antimicrobial properties, fluoride release and inhibition of enamel demineralization.	RMGIC formulations modified with CHX (1.25–2.5%) and TMP (7–14%) were assessed by their antimicrobial activity (agar diffusion, biofilm assay), fluoride/TMP release (15 days), mechanical properties (CS-compressive strength, DTS-diametral tensile) and demineralization tests (fluoride and TMP release, pH-cycling, enamel and cross-sectional hardness).	CHX significantly improved antibacterial and antibiofilm activity. TMP increased fluoride release and reduced enamel demineralization. The highest fluoride release was observed with 14% TMP combinations.	The combination of low CHX (1.25%) and high TMP (14%) provided the optimal balance between bioactivity and prevention of enamel demineralization with little impact on mechanical properties.
Allam et al. [51]	The assessment of the effect of chicken eggshell powder on mechanical properties and ion release of conventional GIC.	In this in vitro study, conventional GIC was modified with 3% and 5% eggshell powder. (groups: A = control, B = 3%, and C = 5%). Tests included compressive strength, microhardness, and fluoride and calcium release at 7 and 30 days.	Addition of eggshell powder increased compressive strength and microhardness. On day 7 calcium release was highest in group C, whereas calcium release was highest in Groups A and B on day 30. Overall, release of calcium and fluoride was not impacted by the addition of eggshell powder.	Eggshell powder improves mechanical properties and maintains or enhances ion release of GIC.
Kirthika et al. [45]	The study evaluated how the incorporation of CPP-ACP, bioactive glass (BAG), chitosan (CH), and MDPB into GIC affected compressive strength, flexural strength, bacterial adhesion and fluoride release.	Conventional GIC was modified by incorporating BAG-, CH- and CPP-ACP into the glass powder, while MDPB was added to the liquid component. Specimens were fabricated using custom-designed molds, while flexural and compressive strength were determined with a universal testing machine. Fluoride release was assessed by UV spectrophotometry after 24 h and 7 days. Colorimetric analysis was employed to determine the extent of bacterial adhesion involving *Streptococcus mutans* and *Lactobacillus acidophilus.*	CH-GIC showed the highest compressive and flexural strength. CPP-ACP-, BAG-, and CH-modified GICs significantly improved mechanical properties compared with conventional GIC. MDPB increased flexural strength without affecting compressive strength. All modified groups showed higher fluoride release at 24 h; BAG-GIC exhibited the highest release. MDPB-GIC demonstrated the lowest bacterial adhesion against *L. acidophilus* and *S. mutans*.	The addition of BAG, CPP-ACP and CH enhanced the mechanical performance of conventional GIC, while MDPB increased resistance to bacterial adhesion without compromising fluoride release.
Bilić-Prcić et al. [110]	The present study investigates the impact of incorporating marine-derived hydroxyapatite (HA) on the surface roughness, microhardness, and fluoride release of conventional and resin-modified glass ionomer cements (GICs).	Fuji II LC and Fuji IX GP Extra (GC Corporation, Tokyo, Japan) were modified with 2, 5, and 10 wt% HA derived from cuttlefish bone. Microhardness, surface roughness, and fluoride release (24 h, 7 days, 45 days) were evaluated using Vickers testing, profilometry, and ion-selective electrode analysis.	Fuji II modified with 10 wt% HA showed significantly higher microhardness. HA incorporation increased surface roughness in both GICs. Fuji IX demonstrated higher fluoride release than Fuji II regardless of modification. Addition of 2 and 5 wt% HA significantly increased fluoride release at all evaluated time points.	Addition of marine-derived HA did not improve surface roughness. HA increased microhardness only in Fuji II with 10 wt% HA. Incorporation of 2 and 5 wt% HA enhanced fluoride release, suggesting that optimal HA concentration may improve chemo-mechanical properties of GICs.
Kumar et al. [44]	To examine the impact of incorporation of nanochitosan particles into conventional glass ionomer cement (GIC) on its mechanical properties and fluoride release, and to compare the modified cement (NCH-GIC) with conventional GIC (C-GIC).	Nanochitosan (10 wt%) was incorporated into conventional GIC powder to produce NCH-GIC. Samples of both C-GIC and NCH-GIC were manufactured and examined for compressive strength, flexural strength, wear resistance, ion release, fluoride release, and surface morphology. Compressive strength was measured at 1 h and 24 h, while fluoride release was evaluated at 1 h, 24 h, and 7 days. Statistical analysis was performed using ANOVA, paired *t*-tests, and Tukey-HSD tests.	NCH-GIC demonstrated significantly higher compressive strength and flexural strength compared with conventional GIC. The flexural strength of NCH-GIC was 21.26 MPa, while C-GIC showed 12.67 MPa. Wear resistance was significantly greater in NCH-GIC due to enhanced bonding between the glass particles and polymer matrix. Fluoride release was consistently higher in NCH-GIC at all evaluated time points up to 7 days. Increased aluminum ion release at 1 h suggested earlier formation of aluminum polysalts in NCH-GIC, which may contribute to enhanced mechanical properties.	In conclusion, incorporation of nanochitosan into conventional glass ionomer cement improved compressive strength, flexural strength, wear resistance, and fluoride release.
AlMatar et al. [111]	The objective of the study was to determine the influence of silver and zinc oxide nanoparticle incorporation into resin-modified glass ionomer cement on microhardness and fluoride release during 14 days of observation.	The resin-modified glass ionomer cement Fuji PLUS (Tokyo, Japan) was used. RMGIC specimens were prepared with 5 wt% silver nanoparticles, 5 wt% zinc oxide nanoparticles, or a combined 1:1 mixture of both nanoparticles at a total concentration of 5 wt%.	RMGIC specimens containing 5% ZnONP and the combined 5% SNP + 5% ZnONP formulation showed noticeable changes in surface ultrastructure, including the presence of surface pores. Fluoride release was highest in the RMGIC modified with 5% SNP + 5% ZnONP compared with the control group and the RMGIC containing only 5% SNP. A color change was also observed in the RMGIC specimens incorporating 5% SNP.	The combined incorporation of SNP and ZnONP into RMGIC enhanced fluoride release and maintained color stability, although it reduced surface microhardness due to pore formation. These findings suggest that dual nanoparticle reinforcement may improve selected properties of RMGIC and may have clinical relevance.
Osinaga et al. [112]	The objective of the present study was to evaluate the influence of ZnSO_4_ incorporation on the ion release, physical properties, and antibacterial activity of conventional cement and RMGIC.	Vitremer and Ketac-Fil powders were modified with 5% or 10% ZnSO_4_. Solubility and flexural strength were tested according to ISO standards, while zinc and fluoride release/uptake were measured over 15 days before and after recharge. Antibacterial activity against *S. mutans* was assessed using agar diffusion tests, and the results were analyzed by ANOVA.	Increasing ZnSO_4_ concentration led to higher solubility, but the values remained below the ISO limit. Flexural strength was not influenced by ZnSO_4_ addition, and Vitremer showed better performance than Ketac-Fil. The greatest zinc release occurred in Vitremer with 10% ZnSO_4_, while fluoride release was similar in both materials and highest during the first 24 h. Antibacterial activity against *S. mutans* was observed only in fresh specimens with the highest ZnSO_4_ content.	The incorporation of zinc reduced microbial growth and enhanced fluoride release, while not causing significant changes in flexural strength or solubility of the tested materials.

**Table 2:** Detailed characteristics of the included studies.

Study	Material	Additive	Fluoride Assessment	Fluoride-Related Findings	Other Findings
Jordão et al. [95]	self-curing GIC: Ketac Molar, Fuji IX	Graphene powder	By ion-selective electrode storage in deionized water, time points: 24 h; 7; 14; 21; 28 days; solution was refreshed between intervals.	peak release at 7 days higher fluoride release at 21 days in graphene groups (1–5%), as well as enhanced long-term release	- increased microhardness (especially 1–2% graphene) - reduced surface roughness - decreased radiopacity - no impact on water sorption/solubility
de Morais Sampaio et al. [96]	Meron (VOCO) Riva (SDI)	ethanolic extract of red propolis (EERP)	By ion-selective electrode, single time point at 24 h, storage at 37 °C.	Fluoride releases unaffected by addition of propolis	- 25% EERP showed best mechanical properties, higher concentrations may worsen properties; - strong antimicrobial activity against *S. mutans* and *C. albicans* (25–50%) - reduced biofilm viability
Patel et al. [97]	Resin-modified glass ionomer cement (RMGIC)	chitosane	By ion chromatography, storage: artificial saliva, time points: 15 and 30 days.	Increased fluoride release in CH group (+8.47% at 15 days; +39.68% at 30 days). Enhanced sustained release.	- adhesive bond strength unchanged - mechanical properties preserved
Peres Fernandes et al. [98]	GC Fuji Ortho LC (RMGIC)	nano-sized sodium trimetaphosphate and/or phosphorylated chitosan	By ion-selective electrode, using pH-cycling model (demineralizing/remineralizing solutions). Analysis of 15 days with daily collection.	- higher fluoride release in TMPnano groups, - similar among modified groups, -improved fluoride availability under cariogenic conditions	- no overall significant effect on mechanical properties (optimal: TMPnano + 0.25% Chi-Ph); - initial reduction in tensile strength (24 h) with recovery at 7 days. - comparable surface hardness between groups; - strong antimicrobial and antibiofilm effect- reduced *S. mutans* viability - non-cytotoxic - potential remineralization effect
Albasso et al. [42]	- Glass-ionomer cement (Medifil, PROMEDICA)	Seashell nanoparticles (5 wt% and 10 wt%)	The fluoride release was evaluated at 1 and 4 weeks in samples stored in deionized water at 37 °C using a fluoride ion-selective electrode.	The addition of seashell nanoparticles significantly enhanced fluoride release (*p* ≤ 0.05), with the highest values observed in the 10% group (10.35 ± 1.46 ppm at week 1; 11.79 ± 0.34 ppm at week 4). No significant difference was found between the 5% and 10% groups at week 1. Fluoride release increased over time in all groups (week 4 > week 1), suggesting improved remineralization potential.	- the addition of seashell nanoparticles significantly improved compressive strength and microhardness, with the best results observed for the 10% group (93.09 ± 0.92 MPa for compressive strength and 55.72 ± 1.19 MPa for microhardness)
Yi et al. [99]	- GC Ortho LC (commercial RMGIC) - PE and PEHB resins	- GC Ortho LC (control; commercial RMGIC) - 40% PE resin + 0% nCaF_2_ + 60% glass (PE control) - 40% PE resin + 20% nCaF_2_ + 40% glass (PE + 20% nCaF_2_) - 40% PE resin + 30% nCaF_2_ + 30% glass (PE + 30% nCaF_2_) - 40% PEHB resin + 0% nCaF_2_ + 60% glass (PEHB control) - 40% PEHB resin + 20% nCaF_2_ + 40% glass (PEHB + 20% nCaF_2_) - 40% PEHB resin + 30% nCaF_2_ + 30% glass (PEHB + 30% nCaF_2_)	Fluoride ion release was measured using an ion-selective electrode in lactic acid solution (pH 4.0) over 70 days, followed by recharge (5000 ppm NaF) and re-release cycles.	The incorporation of nCaF_2_ significantly increased fluoride release and recharge capacity (*p* < 0.05). The highest fluoride release and re-release were observed in the PEHB + 30% nCaF_2_ group, with long-term release rates approximately 110% higher than the commercial RMGIC control. Increasing nCaF_2_ content from 20% to 30% significantly enhanced fluoride release and re-release. Fluoride re-release remained sustained for at least 7 days after each recharge and did not decrease over multiple cycles.	- the addition of nCaF_2_ did not adversely affect shear bond strength - after demineralization, PEHB + nCaF_2_ groups showed bond strength comparable to the commercial RMGIC control; - the materials exhibited good biocompatibility, with cell viability exceeding 70%. PEHB + nCaF_2_ groups showed viability comparable to the control, while PE + nCaF_2_ groups exhibited slightly higher values at early time points.
Li et al. [100]	- Ketac Cem μ (glass ionomer cement) - GC Fuji Ortho LC (resin-modified glass ionomer cement) - Zinc Cement Improved (zinc phosphate cement) - Transbond XT (resin)	Sodium fluoride (NaF) incorporated at different concentrations: - Ketac Cem μ: NaF (7.7% vs. 0%) - GC Fuji Ortho LC: NaF (15% vs. 0%) - Zinc Cement Improved: NaF (10% vs. 0%) - Transbond XT: NaF (38% vs. 0%)	- Ion chromatography (DX 100; Dionex, Camberley, Surrey, UK; suppressed conductivity) - Storage in deionized water at 37 °C - Daily measurements (week 1) and weekly up to 10 weeks - Fluoride expressed as μg/mg or μg/bracket/day	Fluoride release was highest initially and decreased over time. The resin with 38% NaF showed the highest release during the first 2 weeks (*p* < 0.05), followed by a sharp decline. After ~4–5 weeks, RMGIC with 15% NaF exhibited significantly higher and more stable fluoride release compared with other materials (*p* < 0.05). Sustained fluoride release was observed over 10 weeks, with RMGIC showing the most consistent long-term profile.	Not assessed
Pourhajibagher et al. [101]	- GC Fuji Ortho LC (RMGIC)	Ultrasound-activated nanosonosensitizers: n-Cur, n-Emo, and n-Qct (0, 2, 5, 10 wt%)	- Storage in artificial saliva at 37 °C - Measurement at 1, 7, 15, and 30 days - Fluoride measured using pH/ion meter	Fluoride release increased over time for all groups. The addition of nanosonosensitizers did not significantly affect fluoride release compared with the control (*p* > 0.05). No significant differences between experimental groups were observed at any time point.	- shear bond strength decreased with increasing nanoparticle concentration - high concentrations (10% n-Emo, 10% n-Cur, 5–10% n-Qct) showed significantly lower values; the 2% n-Qct, 5% n-Cur, and 5% n-Emo formulations showed less pronounced SBS reductions than the corresponding higher-concentration formulations - setting time decreased after nanoparticle incorporation, with the shortest time observed for 5% n-Emo - ARI scores showed no significant differences between groups. - significant antibiofilm effect against *S. mutans* after ultrasound activation - the lowest bacterial counts were observed for 5% n-Emo and 0.2% CHX - all nanosonosensitizers reduced biofilm by ≥3 log_10_ CFU/mL compared with control.
Lin et al. [102]	- GC Fuji Ortho LC (resin-modified glass ionomer cement)	- Nano-fluorapatite (nano-FA) and nano-fluorohydroxyapatite (nano-FHA) (2.5, 10, 15, 20, 25, 30 wt%; optimal 25 wt%)	- Ultraviolet spectrophotometry - Storage in artificial saliva at 37 °C - Measurements at days 1, 2, 3, 7 and weekly up to 70 days	Fluoride release showed an initial burst followed by a decrease and a subsequent increase over time. The highest release was observed for 25 wt% nano-FA and nano-FHA, with nano-FA providing the most consistent and highest long-term fluoride release. Fluoride release at 70 days was nearly three times higher than the control for nano-FA groups.	- shear and micro-tensile bond strength decreased after nanoparticle incorporation. - GC showed the highest values (SBS: 10.36 ± 3.63 MPa), while GFA (6.58 ± 2.52 MPa) and GFHA (7.72 ± 2.85 MPa) were significantly lower - micro-tensile bond strength followed a similar trend (GC: 22.4 ± 4.4 MPa; GFA: 21.6 ± 3.9 MPa; GFHA: 18.8 ± 5.7 MPa).
Parizi et al. [103]	GC Fuji Ortho LC	silibinin at four concentrations (0%, 1%, 2%, and 5%) mixed into liquid component of RMGIC	- pH/ion meter - samples immersed in 5 mL of artificial saliva at 37 Celcius-test lasted 30 days total - measuring points at 1, 7, 15, and 30 days	- typical initial burst effect in all groups followed by gradual steady release, therefore addition did not disrupt fluoride release model - at day 2 the fluoride release was the significantly higher in sample with 2% silibinin than sample with 1% and control sample - at day 15 fluoride release from all silibinin-containing samples was substantially higher than that of the control sample - at day 30 no significant differences among sample groups - highest cumulative release at the end of the test was observed in a sample with 5% silibinin.	- silibinin caused a dose-dependent reduction in shear bond strength; SBS findings were interpreted relative to the unmodified control under the experimental conditions used. - addition of silibinin did not negatively affect debonding behavior or failure pattern (no significant differences in ARI scores in all tested groups) - silibinin-containing samples significantly reduced bacterial counts in comparison with control, with 5% silibinin samples showing the highest antibacterial potency in test groups - CHX still exhibited superior antibacterial ability than any silibinin test samples - addition of silibinin visibly lessened the biofilm formation, with the best result in 5% sample - none of the silibinin concentrations decreased viability of human gingival fibroblasts significantly, therefore all groups were considered non-cytotoxic
Dobrzynski et al. [88]	GC Fuji Ortho LC	Nanofluorapatite in concentrations of 2% and 5% at 9 different solutions: 1. deioned H_2_O 2. saline solution 3. artificial saliva (AS) pH 4.5 4. AS pH 5.5 5. AS pH 6.0 6. AS pH 7.0 7. AS pH 7.5 8. AS with calcium ions pH 4.5 9. AS with calcium ions pH 5.5	Fluoride release was determined using an ORION Model 9609 ion-selective electrode (Thermo Fisher Scientific Co., Waltham, MA, USA) connected to a CPI-551 Elmetron pH/ion meter. - samples submerged in liquid test solutions at 37 Celcius - test lasted 84 days in total - assessment time points: 1, 3, 24, 48, 72, 96, 168, 336, 504, 672, 840, 1008, 1176, 1344, 1512, 1680, 1848, and 2016 h	Not assessed	Not assessed
Raghimi et al. [104]	- GC Fuji II LC - BracePaste composite GC Fuji II LC reinforced with additive	RMGIC formulations containing Ag/HA/Si hybrid nanoparticles at concentrations of 0.1–2 wt%	- digital ion-selective electrode meter - samples submerged in distilled water at 37 Celcius - test lasted 28 days in total - measuring points 1, 2, 3, 7, 14 and 28 days	Addition of nanoparticles expressed statistically significant increase in fluoride release (*p* < 0.05) in concentration-dependent trend, with highest release noted in 2% Ag/NA/Si additive group.	- mechanical competence of the material was not significantly altered, - in every group compressive strength was in clinically accepted threshold. - the mechanical strength gradually improved with the rising concentration of Ag/HA/Si additive, reaching its peak at 1%, and at 2% it decreased below the outcome of every other sample group (including control group without additive) suggesting that higher concentration may overload the matrix. - the effect is presumed to be linked to silver and silica nanoparticles. - indirect biological benefits (antimicrobial, antibiofilm effect, remineralization potency) are presumed to occur due to added silver and hydroxyapatite nanoparticles, though not directly tested and confirmed.
Salmerón-Valdés et al. [105]	type I glass ionomers: - Conventional glass ionomer Ketac Cem, (3M ESPE, MN, USA) - resin-modified glass ionomer FO (Fuji Ortho, GC CORPORATION, Tokyo, Japan)	A quantity of one gram of halloysite nanotubes (HNs) was modified with 2% chlorhexidine (CX), at concentrations of 5% and 10% relative to the total weight of the powder used to construct each sample.	Not assessed	Not assessed	- incorporation of 5% and 10% HNs + CX did not significantly affect microhardness or setting time of Fuji Ortho LC and Ketac Cem. - addition of CX + HNs significantly increased compressive strength in both cements. - modified ionomer cements showed antibacterial activity against *S. mutans*, whereas control groups showed none. - increasing the concentration from 5% to 10% enhanced the antibacterial effect, with Ketac Cem + 10% CX + HNs showing the greatest inhibition. At 5% concentration, Fuji Ortho LC and Ketac Cem exhibited similar antibacterial activity. - the antibacterial effect was short-term and not maintained beyond 72 h.
Yi et al. [106]	(Transbond XT (termed as TB control) GC Ortho LC (control)	GC + 5% nCaF_2_; GC + 10% nCaF_2_; GC + 20% nCaF_2_; GC + 30% nCaF_2_; GC + 1% DMAHDM; GC + 2% DMAHDM; GC + 3% DMAHDM; GC + 4% DMAHDM	Fluoride and calcium ion release were evaluated from light-cured cement specimens measuring 2 × 2 × 12 mm. 3 specimens per group were immersed in 50 mL sodium chloride solution buffered at pH 4.0, 5.5, or 7.0. Ion concentrations were measured at 1, 3, 7, 14, 21, 28, 35, 42, 49, and 56 days. Fluoride release was assessed using a selective electrode, and calcium release was measured spectrophotometrically.	Addition of nCaF_2_ enhanced fluoride release from the material. DMAHDM did not negatively affect fluoride-releasing capacity. Acidic conditions increased fluoride ion release, suggesting pH-responsive fluoride release. The material maintained fluoride release despite DMAHDM addition and showed enhanced fluoride release with nCaF_2_, particularly under acidic conditions.	- TB control showed the highest enamel bond strength. - Incorporation of nCaF_2_ up to 20% and DMAHDM up to 3% did not produce the marked SBS reductions observed with 30% nCaF_2_ and 4% DMAHDM, respectively. - the combined GC + 20% nCaF_2_ + 3% DMAHDM formulation maintained bond strength comparable to the GC control. - modified GC cements significantly reduced 2-day biofilm viability, metabolic activity, polysaccharide production, and lactic acid production. - the combined formulation showed the strongest antibiofilm effect, with mostly dead bacteria and the lowest metabolic and acidogenic activity. Addition of nCaF_2_ and DMAHDM significantly reduced CFU counts of total microorganisms, total *streptococci*, and *mutans streptococci*. - DMAHDM reduced CFU by nearly 3 log units, while the combined formulation achieved >3 log reductions compared with the GC control. - human gingival fibroblast viability remained acceptable, indicating good cytocompatibility of the modified cements.
Ma et al. [107]	Transbond XT (TB), RMGIC (GC Ortho LC)	2-methacryloyloxyethyl phosphorylcholine (MPC); dimethylaminohexadecyl methacrylate (DMAHDM); nanoparticles of amorphous calcium phosphate (NACP)	Not assessed	Not assessed	- modification of RMGIC with MPC, DMAHDM and NACP maintained enamel shear bond strength. - TB control had numerically higher SBS than GC control, but the difference was not significant. - although modified RMGIC groups maintained SBS comparable to unmodified RMGIC, their SBS was lower than TB control. - MPC/DMAHDM/NACP-modified RMGIC preserved bonding performance relative to GC control but did not reach the SBS level of TB control. - RMGIC modified with MPC, DMAHDM and NACP provided superior protection against enamel prism dissolution caused by biofilm acids compared with unmodified RMGIC and TB control, indicating enhanced resistance to enamel demineralization
Li et al. [108]	- Transbond XT - Transbond Plus - the reinforced glass ionomer cement (GC Fuji ORTHO LC; GC Corporation, Tokyo, Japan)	The nanosilver-containing cement (NSC), nano silver base inorganic antibacterial powder (AgNaZr_2_ (PO_4_)_3_·H_2_O, AGP-ZP003 Shanghai Huzheng Nano Technology Co silver content = 3% + GC Fuji ORTHO LC with the weight ratio of 1:99, 3:97, 5:95, 10:90 and 15:85, respectively, named NSC1, NSC2, NSC3, NSC4 and NSC5	Not assessed	Not assessed	- reported SBS values were approximately 6–8 MPa under the experimental conditions used; bond strength decreased with increasing nanosilver content, and 15% NSC5 showed significantly lower SBS than the control. - fresh NSCs and ORTHO LC exhibited similar antibacterial activity (~8 mm inhibition zones), mainly due to fluoride release; nano-silver powder alone produced a 13 mm inhibition zone. - no inhibition zones were observed after 2 days to 2 weeks of aging, indicating limited long-term antibacterial activity. In the direct contact test, all fresh silver nanoparticle-containing materials completely inhibited bacterial growth, whereas Transbond XT showed no antibacterial effect. - aging progressively reduced antibacterial efficacy; NSC5 retained activity the longest (up to 6 weeks), but no material remained effective after 8 weeks. The antibacterial action of NSCs was mainly attributed to direct contact with cariogenic streptococci rather than silver ion release.
da Silva et al. [109]	Resin-modified glass-ionomer cement (Fuji II LC)	CHX (1.25–2.5%) TMP (7–14%)	Ion-selective electrode, daily measurement over 15 days	Initial burst release followed by stabilization. TMP enhanced fluoride release and enamel fluoride uptake.	- high CHX (2.5%) and TMP (14%) decreased compressive and tensile strength, but lower concentrations maintained acceptable mechanical properties of the material. - strong antibacterial activity, reduced biofilm metabolism.
Allam et al. [51]	Conventional glass-ionomer cement (AquaCem)	Chicken eggshell powder (3%, 5%)	Ion-selective electrode; measurements at 7 and 30 days	Higher fluoride release in B (3%) group; overall release decreased over time; no difference from additive.	- significant improvement in compressive strength and surface hardness (mostly in group C).
Kirthika et al. [45]	Conventional GIC (GC India Dental Pvt. Ltd., India)	CPP-ACP, BAG, CH, MDPB	Fluoride release was evaluated by UV spectrophotometry using Eriochrome cyanide reagent (Medilab Exports Consortium, Haryana, India) after storage in deionized water at 24 h and 7 days.	- all modified groups showed higher fluoride release at 24 h - BAG-GIC showed the highest fluoride release - CPP-ACP- and BAG-GIC remained comparable to control at 7 days - MDPB did not adversely affect fluoride release	- CH significantly improved flexural and compressive strength - CPP-ACP, BAG, and MDPB improved flexural strength - MDPB had no effect on compressive strength - C-GIC showed highest bacterial adhesion - MDPB-GIC showed the lowest adhesion against *L. acidophilus* and *S. mutans* - BAG, CPP-ACP and CH reduced bacterial adhesion compared with control
Bilić-Prcić et al. [110]	Conventional GIC (Fuji IX GP Extra) and resin-modified GIC (Fuji II LC)	Marine-derived hydroxyapatite (2, 5, 10 wt% HA)	Fluoride release was measured using an ion-selective electrode after storage in deionized water at 24 h, 7 days, and 45 days.	- Fuji IX showed significantly higher fluoride release than Fuji II - fluoride release decreased over time - 2 and 5 wt% HA significantly increased fluoride release at all time points - 10 wt% HA increased fluoride release compared with control, but less than 2 and 5 wt% HA groups	- Fuji II with 10 wt% HA showed highest microhardness - HA incorporation increased surface roughness in both GICs - microhardness decreased in most Fuji IX groups with increasing HA concentration -no biological assessment performed
Kumar et al. [44]	Conventional GC Fuji II GIC	Nanochitosan particles, (10 wt%) added to GIC powder	Fluoride release was measured using SPADNS spectrophotometry after submerging the samples in distilled water at 37 °C at 1 h, 24 h and 7 days.	Fluoride release increased over time in both groups but was significantly higher for GIC with added nanochitosan at all time points up to 7 days compared with conventional GIC, which was associated with increased aluminum ion release and a possible catalytic effect of nanochitosan on fluoride particles dispersion.	- GIC with addition of nanochitosan particles exhibited significantly improved compressive strength, flexural strength, and wear resistance compared with conventional GIC. - flexural strength increased from 12.67 ± 0.97 MPa (conventional GIC) to 21.26 ± 0.93 MPa (GIC with nanochitosan additive). - improved properties were linked to reduced interfacial tension, enhanced adhesion between glass particles and polymer matrix, and early formation of aluminum polysalts.
AlMatar et al. [111]	Resin-modified glass ionomer cement Fuji PLUS (Tokyo, Japan).	SNP-silver nanoparticles (5 wt%) ZnO nanoparticles (5 wt%)	Fluoride release was assessed by storing specimens in 10 mL of distilled water for 2, 7, and 14 days, with transfer to fresh solution at each interval. The collected storage media were analyzed by HPLC (high-performance liquid chromatography) with UV–vis detection, and only fluoride ion release was reported	The highest fluoride release was recorded in the 5% SNP + 5% ZnONP group. The control and combined SNP + ZnONP specimens showed a gradual increase in fluoride release by day 7, while the 5% ZnONP group exhibited an initial burst release followed by a reduction.	- FTIR analysis showed characteristic functional groups in all specimens and indicated possible interactions between ZnONP and the cement matrix. - Nano-CT revealed heterogeneous porosity in all groups, with larger pores and more visible cracks in specimens containing 5% ZnONP and 5% SNP + 5% ZnONP. Porosity was lower in the 5% SNP group, while ZnONP-containing specimens showed a broader pore size distribution. - Vickers microhardness testing demonstrated that the combined SNP + ZnONP group had the lowest hardness values, whereas the control and 5% ZnONP specimens showed comparable hardness after 14 days.
Osinaga et al. [112]	Vitremer powders. Ketac-Fil powders.	5% ZnSO(4) 10% ZnSO(4)	Fluoride concentration was determined using a fluoride ion-selective electrode (model 96-09, Orion Inc., Boston, MA, USA). Before each measurement, the system was calibrated with standard fluoride solutions. For analysis, 1 mL of TISAB II solution was added to each vial containing 1 mL of artificial saliva.	Both materials released considerable amounts of fluoride, particularly during the first three days, with the highest release observed in specimens containing the greatest ZnSO_4_ concentration. The effect of fluoride recharge was detected only on the first day, mainly in the K0 group.	- flexural strength analysis showed a significant difference between the tested cements. - Vitremer demonstrated a mean flexural strength of 64.0 MPa, which was almost twice as high as that of Ketac-Fil, which reached 29.4 MPa. - the addition of ZnSO_4_ did not significantly affect flexural strength, and no interaction between the main factors was observed. - the greatest inhibition of *S. mutans* growth was observed in 1 h specimens containing 10% ZnSO_4_, with inhibition zones of about 2.2 mm. -no antibacterial effect was found after 15 days, and recharge did not significantly improve inhibition.

**Table 3:** Directional summary of the effects of functional additives evaluated in the included studies.

Additive or Formulation	Fluoride Release or Recharge	Antibacterial or Antibiofilm Activity	Mechanical or Physical Performance	Overall Interpretation	Reference
Graphene	Favorable under selected concentrations and observation periods	No improvement demonstrated	Favorable at low concentrations for microhardness and surface roughness; radiopacity decreased	Mixed, concentration-dependent profile	Jordão et al. [95]
Red propolis extract	Neutral	Favorable, particularly at higher concentrations	Mostly preserved at intermediate concentration; deterioration of selected properties at some concentrations	Antimicrobial benefit without improved fluoride release	de Morais Sampaio et al. [96]
Chitosan	Favorable	Not assessed	Bond strength preserved	Favorable fluoride-mechanical profile; antimicrobial evidence unavailable	Patel et al. [97]
TMPnano with phosphorylated chitosan	Favorable	Favorable	Preserved or improved at the optimal formulation; transient reductions were reported for selected mechanical outcomes	Promising multidomain profile at 14% TMPnano and 0.25% Chi-Ph	Fernandes/Peres Fernandes et al. [98]
Seashell-derived nanoparticles	Favorable	Not assessed	Favorable for compressive strength and microhardness	Favorable fluoride-mechanical profile	Albasso et al. [42]
nCaF_2_	Strongly favorable, including sustained release and recharge/re-release	Not assessed	Bond strength generally preserved under the tested conditions	Promising fluoride-releasing formulation requiring clinical validation	Yi et al. [99]
Sodium fluoride (NaF) in modified bracket-filling materials	Favorable but material- and time-dependent; high early release from the NaF-loaded composite and more sustained release from the NaF-containing RMGIC	Not assessed	Not assessed	Enhanced fluoride delivery; RMGIC showed the most stable long-term release profile, but antibacterial and mechanical outcomes were not evaluated	Li et al. [100]
Ultrasound-activated nanosonosensitizers	Neutral	Favorable after ultrasound activation	Unfavorable, concentration-dependent reduction in SBS	Antibiofilm benefit accompanied by a mechanical trade-off	Pourhajibagher et al. [101]
Nano-fluorapatite and nano-fluorohydroxyapatite	Favorable	Not assessed	Unfavorable for SBS and µTBS; nano-FA performed more favorably than nano-FHA	Improved fluoride release with reduced bonding performance	Lin et al. [102]
Silibinin	Neutral	Favorable and concentration-dependent	Unfavorable, concentration-dependent reduction in SBS	Antibacterial benefit without sustained fluoride improvement	Parizi et al. [103]
Nanofluorapatite	Favorable, particularly for fluoride recharge	Not assessed	Not adequately assessed for comparative mechanical interpretation	Promising fluoride recharge profile	Dobrzyński et al. [88]
Ag/HA/Si hybrid nanoparticles	Favorable for fluoride recharge	Not assessed	Generally neutral for compressive strength	Favorable fluoride profile without substantial mechanical deterioration	Raghimi et al. [104]
CHX-modified halloysite nanotubes	Not assessed	Favorable	Compressive strength improved; microhardness and setting time generally preserved	Favorable antibacterial-mechanical profile; long-term durability uncertain	Salmerón-Valdés et al. [105]
nCaF_2_ with DMAHDM	Favorable	Strongly favorable	Preserved at the optimal formulation; higher additive loading may reduce SBS	One of the most promising multifunctional formulations	Yi et al. [106]
MPC, DMAHDM, and NACP	Not directly assessed as a fluoride-enhancing formulation	Favorable, including protein-repellent and antibiofilm effects	Bond strength preserved relative to RMGIC under the tested conditions	Favorable antibacterial and enamel-protective profile	Ma et al. [107]
Nanosilver	Not assessed	Favorable initially, but activity decreased with aging	Unfavorable at increasing concentrations	Short-term antibacterial activity with limited durability and mechanical trade-off	Li et al. [108]
CHX with TMP	Favorable, mainly owing to TMP	Favorable, mainly owing to CHX	Mixed; deterioration occurred at higher concentrations	Favorable multifunctional profile at optimized concentrations	da Silva et al. [109]
Eggshell powder	Neutral or limited effect	Not assessed	Favorable for compressive strength and microhardness	Primarily a mechanical rather than fluoride-enhancing modification	Allam et al. [51]
CPP-ACP, BAG, chitosan, or MDPB	Favorable for early fluoride release, particularly BAG	Favorable mainly for MDPB	Favorable or preserved, depending on the additive	Additive-specific benefits; results should not be generalized across the group	Kirthika et al. [45]
Marine-derived hydroxyapatite	Favorable at selected concentrations	Not assessed	Mixed: microhardness increased in one material, but surface roughness also increased	Fluoride benefit accompanied by material-dependent physical changes	Bilić-Prcić et al. [110]
Nanochitosan	Favorable	Not assessed	Favorable for compressive strength, flexural strength, and wear resistance	Favorable fluoride-mechanical profile	Senthil Kumar et al. [44]
Combined SNP/ZnONP	Favorable	Not assessed	Unfavorable or mixed because of reduced microhardness and increased porosity	Improved fluoride release with compromised matrix integrity	AlMatar et al. [111]
ZnSO_4_	Favorable	Favorable only in fresh specimens at higher concentration	Flexural strength preserved; solubility increased with concentration	Initial antibacterial and fluoride benefit, but limited antimicrobial durability	Osinaga et al. [112]

Note: Favorable indicates an improvement relative to the corresponding unmodified or control material within the same study; neutral indicates no statistically significant or clearly relevant difference; unfavorable indicates deterioration of the evaluated property; mixed indicates that the direction of the effect depended on concentration, material type, time point, or individual outcome; not assessed indicates that the respective domain was not evaluated. These categories summarize the direction of the reported within-study effects and should not be interpreted as a universal ranking of additives because of substantial methodological heterogeneity among the included studies.

**Table 4:** QUIN methodological quality assessment of the included studies.

Authors	Clearly Stated Aims/Objectives	Detailed Explanation of Sample Size Calculation	Detailed Explanation of Sampling Technique	Details of Comparison Group	Detailed Explanation of Methodology	Operator Details	Randomization	Method of Measurement of Outcome	Outcome Assessor Details	Blinding	Statistical Analysis	Presentation of Results	SCORE
Jordão et al. [95]	2	0	2	2	2	1	0	2	0	1	2	2	66.67
de Morais Sampaio et al. [96]	2	0	2	2	2	0	0	2	0	0	2	2	58.33
Patel et al. [97]	2	2	2	2	2	0	2	2	0	0	2	2	75
Peres Fernandes et al. [98]	2	0	2	2	2	0	0	2	0	0	2	2	58.33
Albasso et al. [42]	2	2	2	2	2	1	0	2	0	0	2	2	70.83
Yi et al. [99]	2	0	2	2	2	0	2	2	0	0	2	2	66.67
Li et al. [100]	2	0	2	2	2	0	0	2	0	0	2	2	58.33
Pourhajibagher et al. [101]	2	2	2	2	2	0	2	2	0	0	2	2	75
Lin et al. [102]	2	0	2	2	2	0	2	2	0	0	2	2	66.67
Parizi et al. [103]	2	2	2	2	2	0	2	2	0	0	2	2	58.33
Dobrzynski et al. [88]	2	0	2	2	2	0	0	2	0	0	2	2	58.33
Raghimi et al. [104]	2	0	2	2	2	1	0	2	0	0	2	2	62.50
Salmerón-Valdés et al. [105]	2	0	2	2	2	0	0	2	0	0	2	2	58.33
Yi et al. [106]	2	0	2	2	2	0	2	2	0	0	2	2	66.67
Ma et al. [107]	2	0	2	2	2	0	2	2	0	0	2	2	66.67
Li et al. [108]	2	0	2	2	2	0	2	2	0	0	2	2	66.67
da Silva et al. [109]	2	0	2	2	2	0	2	2	0	0	2	2	66.67
Allam et al. [51]	2	0	2	2	2	0	0	2	0	0	2	2	58.33
Kirthika et al. [45]	2	0	2	2	2	0	2	2	0	0	2	2	66.67
Bilić-Prcić et al. [110]	2	0	2	2	2	0	0	2	0	0	2	2	58.33
Kumar et al. [44]	2	0	2	2	2	0	0	2	0	0	2	2	58.33
AlMatar et al. [111]	2	0	2	2	2	0	0	2	0	1	2	2	62.50
Osinaga et al. [112]	2	0	2	2	2	0	0	2	0	0	2	2	58.33

## Data Availability

No new data were created or analyzed in this study. Data sharing is not applicable to this article.

## Supplementary Materials

The following supporting information can be downloaded at: https://www.mdpi.com/article/10.3390/ma19163393/s1. Table S1. PRISMA 2020 Main Checklist.
